# Exploring common genetic contributors to neuroprotection from amyloid pathology

**DOI:** 10.1093/braincomms/fcac066

**Published:** 2022-03-17

**Authors:** Mabel Seto, Emily R. Mahoney, Logan Dumitrescu, Vijay K. Ramanan, Corinne D. Engelman, Yuetiva Deming, Marilyn Albert, Sterling C. Johnson, Henrik Zetterberg, Kaj Blennow, Prashanthi Vemuri, Angela L. Jefferson, Timothy J. Hohman, for the Alzheimer’s Disease Neuroimaging Initiative

**Affiliations:** 1 Vanderbilt Memory and Alzheimer’s Center, Vanderbilt University Medical Center, 1207 17th Ave S, Nashville, TN 37212, USA; 2 Vanderbilt Genetics Institute, Vanderbilt University Medical Center, Nashville, TN 37212, USA; 3 Department of Pharmacology, Vanderbilt University, Nashville, TN 37232, USA; 4 Department of Neurology, Vanderbilt University Medical Center, Nashville, TN 37232, USA; 5 Department of Neurology, Mayo Clinic, Rochester, MN 55905, USA; 6 Department of Population Health Sciences, University of Wisconsin, School of Medicine and Public Health, Madison, WI 53726, USA; 7 Alzheimer’s Disease Research Center, University of Wisconsin School of Medicine and Public Health, Madison, WI 53792, USA; 8 Geriatric Education and Clinical Center, Wm.S.Middleton VA Hospital, Madison, WI 53705, USA; 9 Department of Neurology, the Johns Hopkins University School of Medicine, Baltimore, MD 21287, USA; 10 Department of Psychiatry and Neurochemistry, Institute of Neuroscience and Physiology, The Sahlgrenska Academy at University of Gothenburg, Mölndal 413 90, Sweden; 11 Clinical Neurochemistry Laboratory, Sahlgrenska University Hospital, Mölndal 413 45, Sweden; 12 Department of Neurodegenerative Disease, UCL Institute of Neurology, London WC1N 3BG, UK; 13 UK Dementia Research Institute at UCL, London WC1E 6BT, UK; 14 Department of Radiology, Mayo Clinic, Rochester, MN 55905, USA

**Keywords:** Alzheimer’s, Amyloid, Genetics, Hippocampus

## Abstract

Preclinical Alzheimer’s disease describes some individuals who harbour Alzheimer’s pathologies but are asymptomatic. For this study, we hypothesized that genetic variation may help protect some individuals from Alzheimer’s-related neurodegeneration. We therefore conducted a genome-wide association study using 5 891 064 common variants to assess whether genetic variation modifies the association between baseline beta-amyloid, as measured by both cerebrospinal fluid and positron emission tomography, and neurodegeneration defined using MRI measures of hippocampal volume.

We combined and jointly analysed genotype, biomarker and neuroimaging data from non-Hispanic white individuals who were enrolled in four longitudinal ageing studies (*n* = 1065). Using regression models, we examined the interaction between common genetic variants (Minor Allele Frequency >0.01), including *APOE*-ɛ4 and *APOE*-ɛ2, and baseline cerebrospinal levels of amyloid (CSF Aβ42) on baseline hippocampal volume and the longitudinal rate of hippocampal atrophy. For targeted replication of top findings, we analysed an independent dataset (*n* = 808) where amyloid burden was assessed by Pittsburgh Compound B ([^11^C]-PiB) positron emission tomography.

In this study, we found that *APOE*-ɛ4 modified the association between baseline CSF Aβ42 and hippocampal volume such that *APOE*-ɛ4 carriers showed more rapid atrophy, particularly in the presence of enhanced amyloidosis. We also identified a novel locus on chromosome 3 that interacted with baseline CSF Aβ42. Minor allele carriers of rs62263260, an expression quantitative trait locus for the *SEMA5B* gene (*P* = 1.46 × 10^−8^; 3:122675327) had more rapid neurodegeneration when amyloid burden was high and slower neurodegeneration when amyloid was low. The rs62263260 × amyloid interaction on longitudinal change in hippocampal volume was replicated in an independent dataset (*P* = 0.0112) where amyloid burden was assessed by positron emission tomography.

In addition to supporting the established interaction between *APOE* and amyloid on neurodegeneration, our study identifies a novel locus that modifies the association between beta-amyloid and hippocampal atrophy. Annotation results may implicate *SEMA5B*, a gene involved in synaptic pruning and axonal guidance, as a high-quality candidate for functional confirmation and future mechanistic analysis.

## Teaser

Seto *et al*., report a novel locus on chromosome 3 that modulates the association between baseline amyloid levels and neurodegeneration. Minor allele carriers of rs62263260 exhibit faster hippocampal atrophy among high brain amyloid burden. Functional annotation links rs62263260 to the SEMA5B gene, which is involved in axonal guidance and neurodevelopment.

## Graphical Abstract

**Graphical Abstract:**
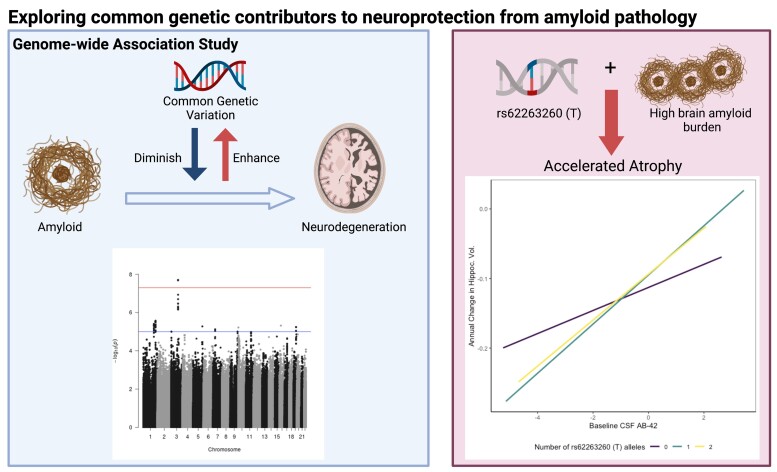

## Introduction

The genomic and phenotypic complexity of Alzheimer’s disease has resulted in a challenging therapeutic landscape including numerous high-profile clinical trial failures and no disease-modifying therapies. Few novel targets have been identified and pursued for Alzheimer’s drug discovery, resulting in the slowed discovery and stalled development of effective treatments.^1–4^ However, recent studies suggest that the exploration of biological mechanisms behind Alzheimer’s disease from a different perspective may allow for new opportunities in Alzheimer’s drug discovery to arise.

Asymptomatic Alzheimer’s disease, or preclinical Alzheimer’s disease, is a phenomenon in which individuals present with the neuropathological hallmarks of Alzheimer’s, but do not yet show clinical signs of cognitive impairment.^5–7^ Some of these individuals may prove to be resilient. Modifiable risk factors that contribute to resilience have been a major focus of the field, including factors like educational attainment that have been leveraged as proxy measures in classical cognitive reserve literature.^8^ Resilience has also been defined in two parts: better than expected cognitive function given the overall level of Alzheimer’s disease pathologies (i.e. cognitive resilience) and less than expected brain atrophy given the level of Alzheimer’s pathologies (i.e. brain resilience).^9^ While modifiable lifestyle factors certainly contribute to such resilience,^10, 11^ there is also emerging evidence from our group and others’ that resilience is heritable and may have a genetic basis.^12–16^

One notable example is the apolipoprotein E (*APOE*) polymorphic alleles, as *APOE-*ɛ2 allele carriers have reduced Alzheimer’s disease risk.^17–19^ In addition, recent studies have suggested that the genetic architecture of resilience is distinct from that of clinical Alzheimer’s disease with only a small contribution of *APOE,*^20^ suggesting that uncovering the genetic architecture of resilience may provide new insight into genomic pathways of protection.

The present analytical approach will further probe the genetic basis of resilience by identifying common genetic variants that modify the association between baseline amyloid deposition and future neurodegeneration.^21–26^ For this study, we will leverage both cerebrospinal fluid (CSF) and positron emission tomography (PET) biomarkers of amyloid-β as well as longitudinal hippocampal volume measured with magnetic resonance imaging (MRI) as our proxy measure of neurodegeneration.^27^

## Materials and methods

### Participants

Data for mega-analysis were acquired from four longitudinal studies of ageing and Alzheimer’s disease that include CSF biomarkers of Alzheimer’s neuropathology, genotype data and neuroimaging. The studies are as follows: the Alzheimer’s Disease Neuroimaging Initiative (ADNI), Vanderbilt Memory and Aging Project (VMAP), Wisconsin Registry for Alzheimer’s Prevention (WRAP) and the Biomarkers of Cognitive Decline Among Normal Individuals (BIOCARD) study. Data from the Mayo Clinic Study of Aging (MCSA) were used for replication. Additional information for each study can be found in the Supplementary Methods.

### Genotyping and quality control procedures

Genotyping was performed by each study on different genotyping platforms (see Supplementary Table 1). Genotyping data were limited to non-Hispanic white individuals whose principal components (PCs) overlayed with individuals of European ancestry using the 1000 Genomes CEU reference panel. Quality control (QC) was performed on genotype data from each cohort separately using PLINK software (version 1.9b_5.2).^28^ Before imputation, single nucleotide polymorphisms (SNPs) with genotyping efficiency <95%, minor allele frequency (MAF) <1%, or deviation from Hardy-Weinberg equilibrium (*P* < 1 × 10^−6^) were excluded. Furthermore, we excluded participants whose call rate was <99%, who exhibited an inconsistency between reported and genetic sex, or who exhibited excess relatedness (PI_HAT > 0.25). We also removed individuals who were outliers based on their ancestral PCs (calculated with EIGENSOFT version 7.2.1)^29^ or who were statistical outliers in heterozygosity rate (>5 SD).

Imputation was performed on the Michigan Imputation Server^30^ using the HRC r1.1.2016 reference panel (Build 37) and SHAPEIT phasing. Imputed genetic data were further filtered for imputation quality (*r*^2^ > 0.9) and biallelic SNPs. To create the joint dataset, we merged genotype data from ADNI, VMAP, WRAP and BIOCARD, excluding multiallelic SNPs, duplicate SNPs, SNPs that were not present in all datasets and SNPs with genotyping efficiency <99%. Additional participants were excluded for relatedness or outlying PCs, resulting in a dataset consisting of 1065 individuals and 5 891 064 variants.

### MCSA GWAS data acquisition, QC and imputation

MCSA GWAS QC procedures are included in Supplementary Methods and described previously.^31^

### Hippocampal volume standardization and slope calculation

MRI was performed at each study site; acquisition and processing protocols are described elsewhere (Supplementary Table 2).^32–35^ We excluded images that failed visual QC, that were taken >90 days prior to CSF acquisition, or that were statistical outliers (>5 SD).

Total hippocampal volume was harmonized across studies using a two-step procedure, and the standardization of all hippocampal volume measurements was based on the first MRI scan of cognitively normal participants at baseline. First, raw hippocampal volume measurements were adjusted to remove the effects of sex and intracranial volume (ICV; see Supplementary Methods). Second, we calculated *Z*-scores using the mean and standard deviation (SD) of the adjusted volume from cognitively normal participants at baseline, resulting in our standardized hippocampal volume variable (Supplementary Fig. 1). Data from ADNI1 and ADNI2 were harmonized separately to account for differences in scanner strength (1.5T vs. 3T).

### MCSA MRI

MRI for MCSA participants was acquired on 3T scanners (General Electric Healthcare, Waukesha, WI, USA) using protocols aligned with ADNI.^36^ Information for acquisition and processing has been described elsewhere.^37–39^ Hippocampal volume and ICV were derived using FreeSurfer (version 5.3).

### CSF biomarker standardization

CSF concentration of the 42 amino acid-long amyloid β form (Aβ42) was acquired via lumbar puncture and quantification by immunoassay performed by each longitudinal ageing study. Acquisition and quantification protocols have been reported by each study.^33–35,40^

CSF Aβ42 was harmonized using a two-component Gaussian mixture model (GMM).^41^ The mean and SD estimated from the model-predicted low amyloid gaussian distribution in cognitively normal individuals was used to standardize all values (Supplementary Fig. 2A) as previously described.^41–43^

### Amyloid positron emission tomography

To support our findings, we leveraged amyloid PET data from MCSA participants measured with Pittsburgh compound B ([^11^C]-PiB), as described elsewhere.^44,45^

We also examined amyloid PET data from ADNI measured with Pittsburgh compound B ([^11^C]-PiB) and florbetapir ([^18^F]-AV-45). Additional details on acquisition and pre- and post-processing pipelines can be found on the ADNI website (www.adni-info.org). Mean standardized uptake value ratio (SUVR) values were standardized using a similar two-component GMM as aforementioned, following previously published methods (Supplementary Fig. 2B).^41,46^

### Statistical analyses

Genome-wide association analyses (GWAS) were conducted using the joint dataset (see above) with PLINK and R (version 3.6.0). Both baseline hippocampal volume and annual change in hippocampal volume were used as continuous outcomes. The annual change in hippocampal volume was determined using linear mixed-effects regression, where the intercept and slope (time from baseline MRI scan) were entered as both fixed and random effects. Covariates for the GWAS included age at first MRI, sex and the first three ancestral PCs (calculated using EIGENSOFT version 7.2.1)^29^ to account for unmeasured population stratification. For computational efficiency, we extracted the hippocampal volume slopes from mixed-effects regression models and entered them as continuous outcomes in a linear regression with PLINK. The interaction term between each SNP and continuous CSF Aβ42 was used to identify variants that modified the association between Aβ42 and annual change in hippocampal volume. All variants were tested using additive coding. Genome-wide significance was set *a priori* to *P* < 5 × 10^−8^.^47^ Although this linear regression approach was more computationally feasible, the full linear mixed-effects model has multiple advantages including the estimation of both intercepts and slopes in the same model. For that reason, we did run the full linear mixed-effects model for all variants meeting suggestive significance (*P* < 1 × 10^−5^) to ensure our results are not driven by the two-stage analytical approach (Supplementary Table 3) and to have a model that aligns with the linear mixed-effects model used in our independent replication. Sensitivity analyses included *APOE-*ɛ4 allele count, MRI scanner strength and a variable for cohort as additional covariates. Additional sensitivity analyses include stratifying by diagnosis, ageing study and adding a cohort × age interaction term (Supplementary Tables 4 and 5).

To validate the candidate locus discovered in our primary analyses, we also tested the target SNP, rs62263260, using additive coding in the independent dataset from MCSA (*n* = 808). Replication analyses used a mixed-effects linear regression to examine the SNP interaction with baseline amyloid PET SUVR, against longitudinal hippocampal volume as the outcome and including age, sex and ICV as covariates. In this model, ICV was included as an additional covariate because hippocampal volume measurements were not adjusted for the effect of ICV in MSCA.

We also leveraged amyloid PET data from ADNI (*n* = 667) testing the SNP interaction with standardized mean SUVR on the same hippocampal outcome. Covariates included age, sex and PET tracer. Both linear and linear mixed-effects regression models were used. Harmonization across tracers was completed leveraging a GMM as previously published.^42^

Finally, we used a linear regression model to assess the interaction between *APOE* allele count (ɛ4 additive coding and ɛ2 dominant coding due to few homozygous carriers) with CSF amyloid on cross-sectional and longitudinal hippocampal volume (*n* = 1537, Supplementary Table 6).

### Functional annotation

Expression quantitative trait locus (eQTL) annotation was performed using the NIH Genotype-Tissue Expression (GTEx) Portal^48^ and brain cortex eQTL data from Sieberts *et al*. When assessing eQTL *P*-values for the 44 available tissues within GTEx, we performed Bonferroni correction to account for multiple comparisons (significant *P* < 0.0011). Additional annotation leveraged both INFERNO (http://inferno.lisanwanglab.org/) and the Brain xQTL Serve database (http://mostafavilab.stat.ubc.ca/xqtl/).

### Colocalization analysis

To examine genes in the region of the significant locus, we performed colocalization analysis using summary statistics from the SNP × CSF Aβ42 GWAS and brain cortex eQTL data from Sieberts *et al*., (i.e. dorsolateral prefrontal cortex, temporal cortex)^49^ as well as eQTL data from GTEx v8 (i.e. tissues where rs62263260 was a statistically significant eQTL for any gene: oesophagus muscularis, testis, brain anterior cingulate cortex BA24). Using coloc (version 3.2-1),^50,51^ we performed colocalization in a 1 Megabase window around the lead SNP, rs62263260 with default priors.^51^ All protein-coding genes within that window (Chromosome 3, 123175327:122175327) were tested (Supplementary Table 7). A posterior probability greater than 80% (PP4 > 0.8) is indicative of colocalization.^50,51^

### Post-hoc SEMA5B analyses

To assess whether *SEMA5B* expression differs by AD diagnosis, we utilized summaries of case/control analyses from the Accelerating Medicines Partnership Program for Alzheimer’s Diseasee (AMP-AD). Data from this project are made freely available online (https://agora.adknowledgeportal.org).

Furthermore, we examined neuronal *SEMA5B* expression data. Pyramidal neuron expression data for these analyses was obtained from the NIH Gene Expression Omnibus (https://www.ncbi.nlm.nih.gov/geo/). Additional details on brain collection, expression profiling and microarray analysis are described elsewhere.^52–55^ Tissues include the entorhinal cortex, hippocampus, medial temporal gyrus, posterior cingulate cortex, primary visual cortex and superior frontal gyrus.

Repeated measures ANOVA was used to evaluate differences in *SEMA5B* expression in AD patients compared to controls across brain regions. Covariates included age, sex and brain region. Post-hoc paired comparisons within each region were performed leveraging independent samples *t*-tests (one-tailed). We corrected for multiple comparisons leveraging the Bonferroni procedure for the six brain regions evaluated.

### MAGMA pathway analysis

Gene and pathway analyses were conducted using MAGMA version 1.08.^56^ Gene test analyses used the SNP-wise mean model specified in MAGMA. Results were corrected for multiple comparisons using the false-discovery rate (FDR) procedure. Gene set consortia are described in Supplementary Methods.

### Data availability

Data from the ADNI study are shared through the LONI Image and Data Archive (https://ida.loni.usc.edu/). Data from BIOCARD can be requested at https://www.biocard-se.org/. Data from WRAP can be requested at https://wrap.wisc.edu/data-requests/. Sieberts *et al*.^49^ brain cortex eQTL data were obtained through the AMP-AD Knowledge Portal. Additional data sharing will be facilitated by the individual cohort study groups.

## Results

Participant characteristics are presented in Table 1. We observed statistically significant differences between participants in each diagnostic category as expected except for the average number of follow-up visits. Participants in the BIOCARD and WRAP studies are younger than those enrolled in ADNI and VMAP (Supplementary Table 8). Additionally, ADNI includes more participants that have been diagnosed with MCI and Alzheimer’s disease than in VMAP, WRAP or BIOCARD.

**Table 1:** Participant characteristics by diagnosis

N	NC	MCI	AD	Total^a^	*P*-value
	490	475	100	1065	
Age at baseline	68.4 ± 9.3	72.5 ± 7.3	74.5 ± 8.4	70.8 ± 8.7	<0.001
Sex, % female	53%	39%	48%	47%	0.002
% *APOE*-ɛ4 carriers	29%	47%	67%	41%	<0.001
% *APOE*-ɛ2 carriers	13%	9%	3%	10%	<0.001
Std. CSF Aβ42	−0.75 ± 1.6	−1.70 ± 1.7	−2.52 ± 1.3	−1.34 ± 1.7	<0.001
Number of Visits	3.46 ± 1.83	4.00 ± 1.86	2.80 ± 1.22	3.64 ± 1.83	0.9
Neuroimaging Measurements (MRI)					
Std. Hippocampal Volume	−0.01 ± 1.0	−0.84 ± 1.3	−2.1 ± 1.3	−0.58 ± 1.3	<0.001
Std. Hippocampal Vol. Slopes	−0.10 ± 0.1	−0.15 ± 0.1	0.21 ± 0.1	−0.14 ± 0.1	<0.001

Analysis of variance (ANOVA) analyses indicated significant differences (*P* < 0.05) across diagnostic groups for all demographic categories except for the average number of visits. Values given are mean ± standard deviation unless otherwise noted.

Abbreviations: NC, normal cognition; MCI, mild cognitive impairment, AD, Alzheimer’s disease; CSF, cerebrospinal fluid; Aβ42, β-amyloid-42

^a^ Consists of participants from ADNI, VMAP, WRAP and BIOCARD.

Using the composite dataset, we performed GWAS to identify common SNPs that modify the association between baseline CSF Aβ42 and baseline hippocampal volume as well as annual change in hippocampal volume. Suggestively significant loci (*P* < 1 × 10^−5^) are displayed in Supplementary Tables 3, 9 and 10. We also expand on a study by Chiang *et al*.^57^ that explored whether *APOE-*ɛ4 allele status modified the association between baseline CSF amyloid and longitudinal changes in hippocampal volume.

### APOE allele associations with hippocampal atrophy

*APOE* results are presented in Table 2. As expected, *APOE-*ɛ4 allele count was associated with lower baseline hippocampal volume (*β* = −0.43, *P* < 2 × 10^−16^) and faster atrophy (*β* = −0.03, *P* < 2 × 10^−16^). Additionally, *APOE-*ɛ2 carriers have greater hippocampal volume at baseline (*β* = 0.25, *P* = 0.02) and slower atrophy (*β* = 0.02, *P* = 0.0002) compared to non-carriers.

**Table 2:** *APOE*-ɛ4 and *APOE*-ɛ2 associations with baseline hippocampal volume

Predictor	Outcome	B	SE	*P*-value	Adj. *r*^2^	Δ*r*^2^
*APOE*-ɛ4^a^	Baseline HV	−0.43	0.05	< 2.00e−16	0.185	0
*APOE*-ɛ4 × CSF Aβ42^b^	Baseline HV	0.11	0.03	0.0004	0.216	3.1
*APOE*-ɛ2^a^	Baseline HV	0.25	0.10	0.0168	0.146	0
*APOE*-ɛ2 × CSF Aβ42^b^	Baseline HV	−0.13	0.06	0.0435	0.201	5.5
*APOE*-ɛ4^a^	Longitudinal HV	−0.031	0.003	< 2.00e−16	0.193	0
*APOE*-ɛ4 × CSF Aβ42^b^	Longitudinal HV	0.0056	0.002	0.0024	0.248	5.5
*APOE*-ɛ2^a^	Longitudinal HV	0.0236	0.006	0.0002	0.140	0
*APOE*-ɛ2 × CSF Aβ42^b^	Longitudinal HV	−0.0054	0.004	0.152	0.235	9.5

Abbreviations: HV, hippocampal volume; B, beta; SE, standard error; Δ*r*^2^; change in *r*^2^; Adj. *r*^2^, adjusted *r*^2^.

^a^ Model: Hippocampal Volume ∼ Age + Sex + ***APOE***

^b^ Model: Hippocampal Volume ∼ Age + Sex + ***APOE*** × **CSF Aβ42**

### APOE allele interactions with baseline CSF Aβ42

**Figure 1:**
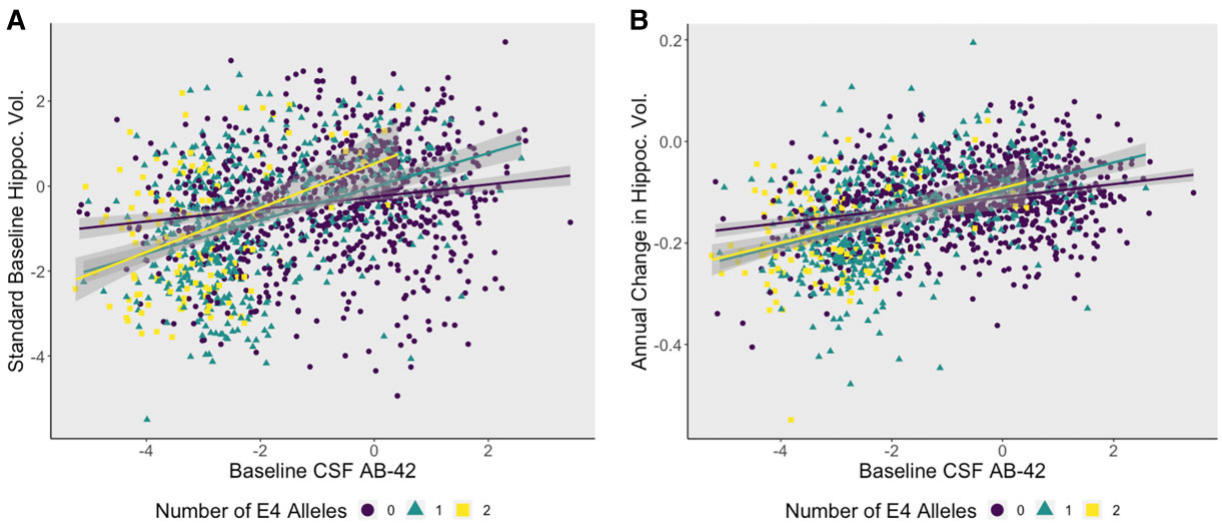
***APOE*-ɛ4 allele carriers have smaller hippocampal volumes at baseline and worse atrophy in the presence of high levels of brain amyloid pathology.** (**A**) A plot demonstrating how *APOE*-ɛ4 allele count modifies the association between Aβ42 and baseline hippocampal volume in a dose-dependent manner (*β* = 0.11, *P* = 0.0004). The *y*-axis represents baseline standardized hippocampal volume, and the *x*-axis represents standardized CSF levels of Aβ42 (*z*-scores). Points and lines are colour coded by genotype, where *APOE*-ɛ4 heterozygotes are denoted by the green line and homozygotes are red. (**B**) *APOE*-ɛ4 positivity increases the rate of atrophy in individuals with high brain amyloid burden (*β* = 0.0056, *P* = 0.0024). There appears to be no change between heterozygous and homozygous carriers of the ɛ4 allele.

As seen previously by Chiang *et al*.,^57^*APOE-*ɛ4 significantly interacted with baseline CSF Aβ42 (*β* = 0.11, *P* = 0.0004, Fig. 1) on hippocampal volume such that *APOE*-ɛ4 carriers with higher brain amyloid burden display lower hippocampal volumes and more rapid hippocampal atrophy. We also observe an interaction between *APOE*-ɛ2 and baseline CSF Aβ42 on baseline hippocampal volume, though it did not survive correction for multiple comparisons. *APOE*-ɛ2 did not interact with CSF Aβ42 on longitudinal change in hippocampal volume (Table 2 and Supplementary Table 1^1^).

### Variant interactions with baseline CSF Aβ42

**Figure 2:**
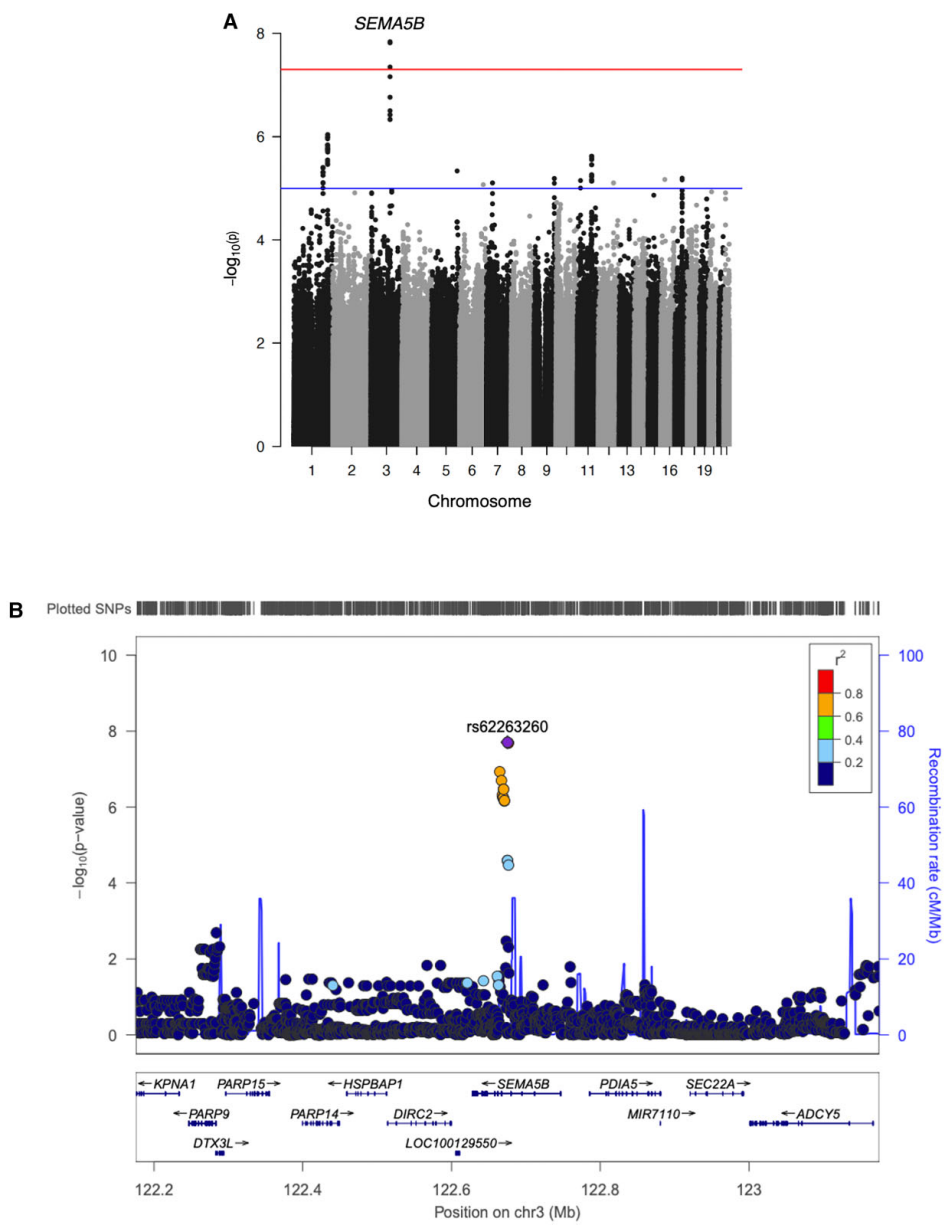
**Three SNPs in an intronic region of the *SEMA5B* gene met genome-wide significance in the SNP × CSF Aβ42 GWAS.** (**A**) The Manhattan plot of the genome-wide association study. The threshold for genome-wide statistical significance (*α* = 5 × 10^−8^) is indicated by the red line. The blue line represents the suggestive threshold for significance (*α* = 1 × 10^−5^). (**B**) A LocusZoom plot of *SEMA5B* and additional genes in the selected 1 Megabase region. Points are coloured by LD with the top variant, where higher *r*^2^ values are coloured in red and lower *r*^2^ values are coloured in blue based off of LD calculated in non-Hispanic whites of European descent. The diamond represents the variant with the smallest *P*-value.

**Figure 3:**
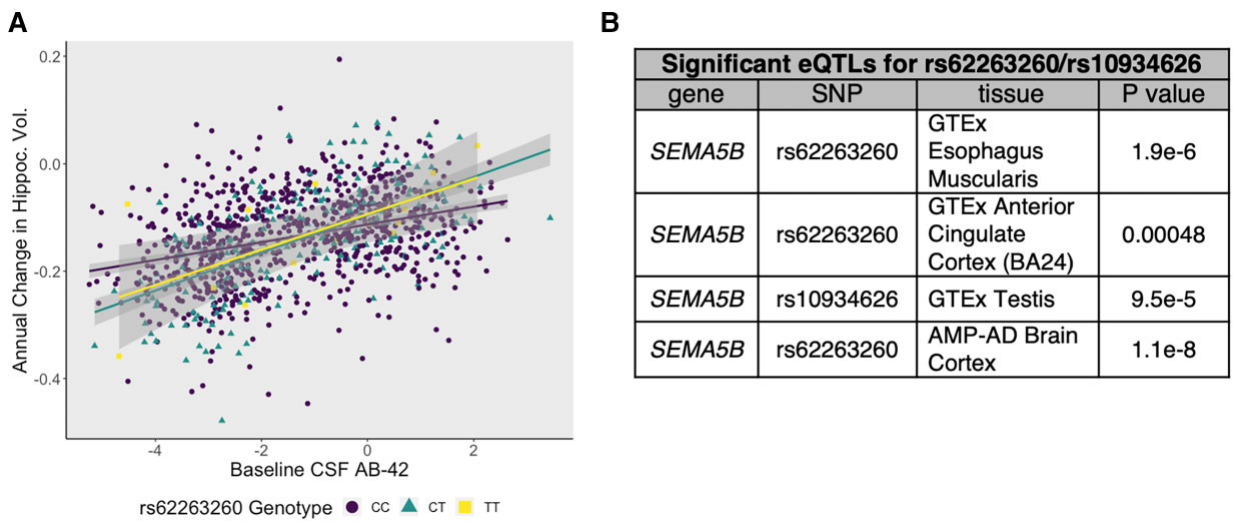
**rs62263260, the index SNP, modifies the association between baseline beta-amyloid and hippocampal atrophy** (**A**) A plot demonstrating how the index SNP, rs62263260, modifies the association between CSF Aβ42 and hippocampal atrophy. The *y*-axis represents annual change in standardized hippocampal volume, and the *x*-axis represents standardized CSF levels of Aβ42 (*z*-scores). Points and lines are colour coded by genotype. Individuals harbouring higher levels of baseline pathology exhibit worse hippocampal atrophy (*β* = 0.026, *P* = 1.46 × 10^−8^). (**B**) Tissues where rs62263260 or rs10934626 (LD *r*^2^ > 0.9) is a statistically significant eQTL for the *SEMA5B* gene.

No significant interactions with CSF Aβ42 in cross-sectional analyses were observed. In longitudinal analyses, we identified a novel genetic locus on chromosome 3 (rs62263260-T, *β* = 0.026, *P* = 1.46 × 10^−8^, MAF = 0.12, Table 3, Supplementary Table 1^2^) that is located within an intron of the *SEMA5B* gene (Figs. 2A and B). Among participants harbouring a high baseline brain amyloid burden (i.e. low CSF Aβ42 levels), minor allele (T) carriers of rs62263260 demonstrated a faster rate of hippocampal atrophy (Fig. 3A). At lower brain amyloid levels, minor allele carriers of rs62263260 had slower rates of hippocampal atrophy. Two additional SNPs within this region reached genome-wide significance (Table 3) and are in high LD (*r*^2^ > 0.8) with the index SNP, rs62263260 (Fig. 2B). The main effect of rs62263260 was not significantly associated with longitudinal atrophy (*P* > 0.1). Genome-wide significance of the rs62263260 × CSF Aβ42 interaction did not change when using linear mixed-effects regression (*β* = 0.03, *P* = 3.13 × 10^−8^) as opposed to linear regression (Supplementary Tables 3 and 13).

**Table 3:** Variant Interactions with CSF β-Amyloid

Variant	chromosome	BP	allele	MAF	B	SE	*P*-value
rs62263260	3	122675327	T	0.121	0.02621	0.0046	1.46e−08
rs11707826	3	122676305	T	0.122	0.02616	0.0046	1.53e−08
rs10934626	3	122676523	T	0.122	0.02616	0.0046	1.53e−08

Abbreviations: BP, base pair; MAF, minor allele frequency; B, beta; SE, standard error.

### Replication of rs62263260 interaction with amyloid load in the Mayo Clinic Study of Aging

In the independent MCSA cohort where amyloid burden was assessed by [^11^C]-PiB PET, rs62263260 again displayed a significant interaction with baseline brain amyloid levels to predict longitudinal hippocampal atrophy (*n* = 808, *β* = −0.24, *P* = 0.0112). The presence of the minor (T) allele was associated with a faster rate of hippocampal atrophy among those with higher baseline amyloid burden (i.e. higher levels of amyloid PET and/or lower levels of CSF amyloid), and slower rates among those with low amyloid burden validating our initial findings in the discovery dataset. Similar results to MCSA were observed when leveraging amyloid PET data from ADNI (*n* = 667; *β* = −0.0055, *P* = 0.0045; Supplementary Fig. 3). Linear mixed-effects regression results (*β* = −0.013, *P* = 0.013) were largely consistent with the aforementioned PET results in ADNI.

### Sensitivity analyses

The rs62263260 × amyloid interaction results maintained genome-wide significance in sensitivity analyses covarying for age, sex, PC1-3, *APOE-*ɛ4 and scanner strength (Supplementary Table 1^3^). When covarying for age, sex, PC1-3 and study, the significance becomes slightly attenuated (*P* = 7.7 × 10^−8^).

### Functional annotation of significant SNPs

The index SNP rs62263260, is a significant eQTL for the *SEMA5B* gene in the brain with associations in other tissues, including the oesophagus (Fig. 3B). In addition, carriers of the minor allele (T) appear to have higher levels of *SEMA5B* expression compared to non-carriers (Supplementary Fig. 4, eQTL information from Sieberts *et al*., 2020). To determine whether *SEMA5B* was the acting gene in the region, colocalization analysis was performed. rs62263260 strongly colocalized with *SEMA5B* expression in the oesophagus muscularis in GTEx v8 (PP4 > 0.99). In other datasets where rs62263260 or its neighbouring SNPs were significant eQTLs for *SEMA5B*, colocalization results were negative (PP3 > 80%) or inconclusive (Supplementary Table 7).

In addition, rs62263260 and SNPs in the surrounding region significantly disrupted six transcription factor binding sites (p.fdr < 0.05, Supplementary Table 14), but were not enriched for enhancer sites and were not methylation-QTLs or histone-QTLs in any queried database.

### Post-Hoc analysis of *SEMA5B* expression in brain

**Figure 4:**
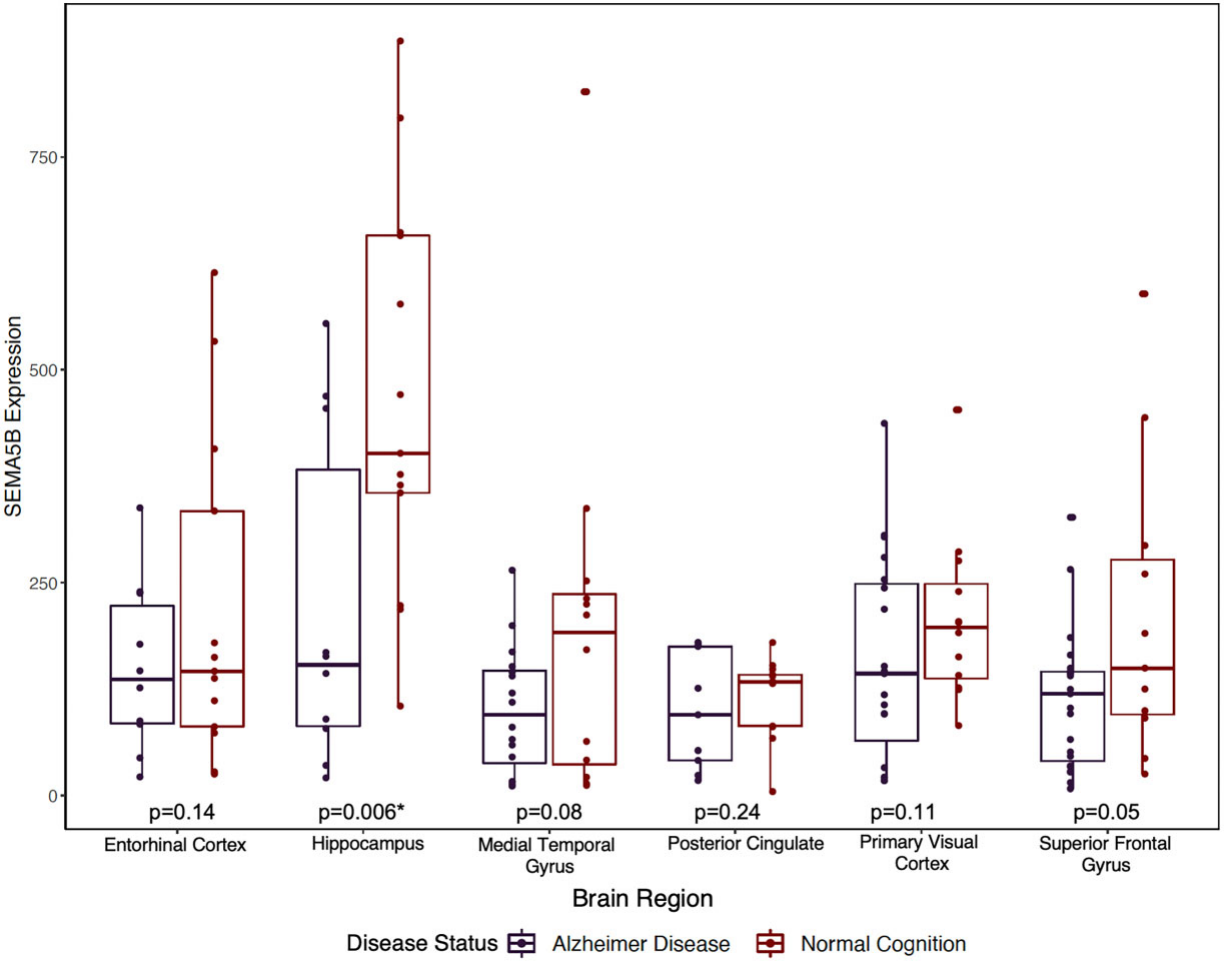
**Hippocampal pyramidal neurons in Alzheimer’s disease brains express less *SEMA5B* than those from cognitively normal controls.** A box plot summarizing laser-captured neuronal expression of *SEMA5B* across brain regions (i.e. entorhinal cortex, hippocampus, medial temporal gyrus, posterior cingulate cortex, primary visual cortex and superior frontal gyrus) in AD cases and controls such that each point represents a sample’s *SEMA5B* expression. Across regions, we observed lower expression of *SEMA5B* in AD compared to controls (*F*(1,152) = 17.45, *P* < 0.0001). In post-hoc paired comparisons, the association was particularly pronounced in the hippocampus surviving Bonferroni correction for multiple comparisons (*P* = 0.006).

Using Agora, a publicly available database powered by the AMP-AD Consortium (https://agora.adknowledgeportal.org/genes/(genes-router: gene-details/ENSG00000082684), we examined whether AD diagnosis had any effect on *SEMA5B* gene expression. In multiple brain tissues, including cerebellum, prefrontal cortex and temporal cortex, *SEMA5B* expression is decreased in AD brains in comparison to controls. To ensure that the differences observed on Agora were not due to cell-type differences in the bulk tissue, we also leveraged a laser-captured neuronal gene expression dataset^52–55^ to assess neuron-specific *SEMA5B* expression differences by diagnosis. Similar to the results seen on Agora, we observed a main effect of diagnosis on *SEMA5B* expression (*F*(1,152) = 17.45, *P* < 0.0001) whereby we observed lower expression of *SEMA5B* in AD compared to control neurons (Fig. 4). When evaluating each region individually in post-hoc paired comparisons, we observed that the difference was particularly pronounced in the hippocampus (*T*(20.768) = −2.79, *P* = 0.006).

### Gene and pathway results

In gene-level analyses, the *TOMM40* interaction with CSF Aβ42 on hippocampal atrophy was the top result (*P* = 1.60 ×10^−5^, p.fdr = 0.28), but did not survive multiple corrections. The *TOMM40* signal was further attenuated when covarying for *APOE* as expected (p.fdr = 0.74).^58^

**Figure 5:**
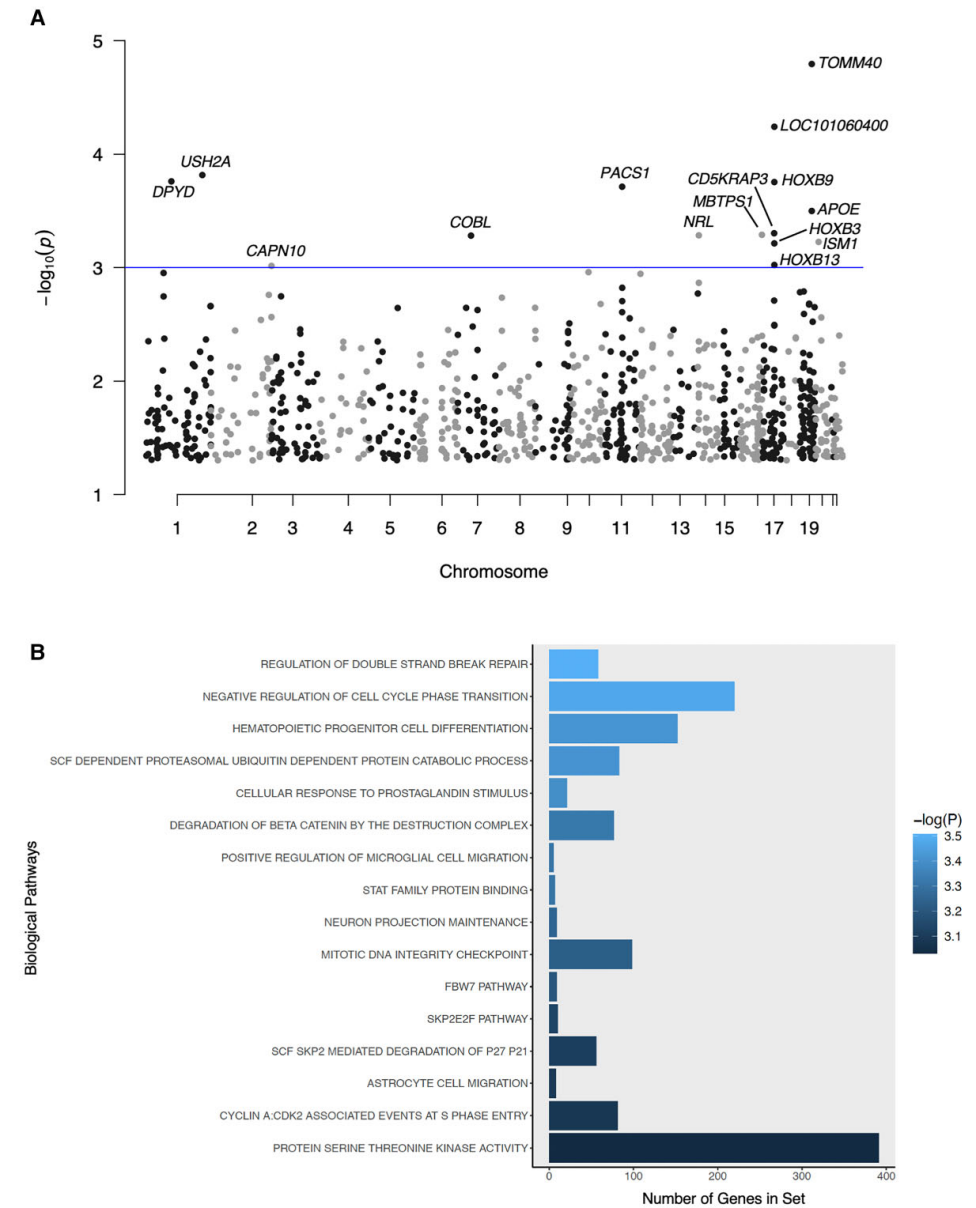
**Summary of nominally significant MAGMA gene- and pathway-level results.** (**A**) A Manhattan plot summarizing chromosome and *P*-value for all genes tested by MAGMA. The threshold for nominal significance is indicated by the blue line (*α* = 1 × 10^−3^). *TOMM40* is the most significant result with a *P*-value of 1.60 × 10^−5^. (**B**) A bar plot summarizing pathway-level results with *P* < 1 × 10^−3^. The *y*-axis represents the number of genes in each pathway gene set. Bars are filled according to *P*-value. The most significant pathway is ‘regulation of double-strand break repair’ (*P* = 3.11 × 10^−4^).

Our top pathway-level results included the GO term ‘regulation of double-strand break repair’ (*P* = 3.11 × 10^−4^) but it did not survive correction. Nominally significant gene- and pathway-level results are reported in Fig. 5 and Supplementary Tables 15–18.

## Discussion

In the current study, we identified a novel locus that modifies the association between baseline CSF Aβ42 and the annual rate of hippocampal volume decline. Specifically, minor allele (T) carriers of rs62263260 exhibit faster rates of hippocampal atrophy among individuals with biomarker evidence of amyloidosis. In contrast, rs62263260 minor allele carriers with low amyloid burden appear to be protected from neurodegeneration compared to non-carriers. Importantly, we observed evidence of this interaction effect across PET and CSF measures of amyloidosis and replicated this interaction effect in an independent dataset. Moreover, our top variant is a strong eQTL for *SEMA5B*, a gene involved in synaptic pruning and axonal guidance. Additionally, we replicated previous work demonstrating that *APOE-*ɛ4 modifies the association between baseline CSF amyloid on both cross-sectional and longitudinal measures of hippocampal volume. Though additional studies are needed, the present results suggest that axonal guidance and synaptic pruning genes, along with *APOE,* may modulate the association between amyloid pathology and downstream neurodegeneration, providing exciting targets for future mechanistic studies.

### Variants on chromosome 3 drive increased susceptibility to amyloid-dependent neurodegeneration

Notably, our top GWAS finding rs62263260 and the additional SNPs within the region have not been linked to Alzheimer’s in any previous case-control studies of clinical Alzheimer’s disease and Alzheimer’s risk.^59,60^ It is also not significantly associated with diagnosis in our study (*P* = 0.47). As in previous studies examining Alzheimer’s disease endophenotypes as outcomes,^61^ rs62263260 may be more related to the rate of disease progression than risk for the onset of clinical disease.

rs62263260 is a significant eQTL for the *SEMA5B* gene in two independent eQTL studies and is colocalized with *SEMA5B* in oesophageal tissue. Though *SEMA5B* expression in oesophageal tissue is not directly linked to neurodegeneration, it should be noted that studies leveraging the NIH GTEx portal have suggested that genetic regulation of gene expression is conserved across many tissues,^62,63^ thus, significant results in seemingly non-relevant tissues, such as the oesophagus, with increased sample size (and subsequently, statistical power), could still provide insights into hypothetical disease processes. However, further study in highly relevant tissues (i.e. hippocampus) is still needed to conclusively elucidate its role in amyloid-related hippocampal atrophy. *SEMA5B* encodes semaphorin 5B (Sema5B), which is expressed in both the developing and adult hippocampus.^48,64–66^ Proteins within the semaphorin family, including Sema5B, facilitate neural development, axonal growth and synapse maintenance.^67^ Sema5B is being actively studied and is not well characterized, but *Sema5b* knockout mice exhibit aberrant neuronal branching and axonal pathfinding defects.^68–71^ In contrast, overexpression of *Sema5b* in mouse hippocampal neurons resulted in a decrease in synapse number.^64^

The direction of the *SEMA5B* association in the present manuscript is difficult to determine, though preliminary eQTL results suggest that the minor allele of rs62263260 is associated with increased expression of *SEMA5B* in tissues including the brain,^49^ oesophagus and testes (Supplementary Fig. 4). Thus, it may be that higher expression of *SEMA5B* is associated with slower hippocampal atrophy in the absence of amyloidosis, but more rapid neurodegeneration in the presence of amyloid. In contrast to the eQTL direction of effect, there is evidence that *SEMA5B* expression is downregulated in Alzheimer’s disease brains as reported by the Agora platform (https://agora.ampadportal.org/genes) and within our post-hoc analyses, further suggesting a change over the course of disease. We hypothesize that *SEMA5B* expression and function may change as Alzheimer’s disease progresses, though further mechanistic study of *SEMA5B* in relevant brain tissues is truly needed to confirm its role and function in neurodegeneration.

### APOE-ɛ4 carriers exhibit increased susceptibility to neurodegeneration in the presence of amyloidosis

*APOE-*ɛ4 is the strongest genetic risk factor for late-onset Alzheimer’s disease, causing a 2- to 3-fold increased risk of Alzheimer’s among heterozygous *APOE-*ɛ4 carriers, and up to a 15-fold increased risk among homozygous *APOE-*ɛ4 carriers.^72,73^*APOE-*ɛ4 increases the pathological deposition and aggregation of Aβ in the brain—even in cognitively normal older adults—and has also shown evidence of independent associations with tau and cerebrovascular disease.^74,75^ Our analyses add to existing literature suggesting that carriers of *APOE*-ɛ4 exhibit faster hippocampal volume decline in the presence of brain amyloidosis. Interestingly, the cross-sectional effects on baseline hippocampal volume appear to occur in a dose-dependent manner. However, we do not see any difference in the association between higher levels of amyloid and neurodegeneration in *APOE*-ɛ4 heterozygotes compared to *APOE*-ɛ4 homozygotes, perhaps suggesting the additional impact of homozygous carriership on hippocampal volume was already present at baseline in these cohort studies. *APOE-*ɛ4 positivity has been associated with accelerated seeding of amyloid pathology and an earlier onset of amyloid positivity.^76,77^ Furthermore, it has been suggested that the length of amyloid positivity correlates positively with the rate of the future progression of disease.^77^ Altogether, the results add to a growing body of literature suggesting that *APOE* contributes to the progression of Alzheimer’s disease both upstream and downstream of amyloidosis.

### Strengths and limitations

This study has multiple strengths including the use of harmonized CSF and PET amyloid values in addition to longitudinal neuroimaging data from well-characterized ageing studies. We were also able to replicate our amyloid results in an independent cohort. In this study, as well as others, we have also demonstrated that our harmonization processes are viable for increasing sample size, laying the foundation for future large-scale genomic discovery analyses of resilience.

However, our study is not without limitations. Our sample was restricted to individuals who were highly educated, non-Hispanic white, and was free of other health comorbidities, limiting the generalizability of our results to additional populations. Though we were able to harmonize and standardize the CSF Aβ42 values and hippocampal volume measurements across cohorts, subtle differences still remain possible due to differences in age and enrollment criteria (Supplementary Table 4). Additionally, as our results are based on cross-sectional amyloid data, we cannot exclude that parts of our findings could be explained by the recent suggestion that *APOE* genotype could be used as a surrogate measure of time with Aβ pathology,^78^ i.e. that Aβ-positive *APOE*-ɛ4 carriers have had Aβ pathology 10–15 years longer than Aβ-positive non-carriers, and that they therefore are further along in the neurodegenerative phase of Alzheimer’s disease. This hypothesis needs to be addressed in future longitudinal studies.

Looking forward, further efforts to harmonize biomarker and neuroimaging data from additional cohorts will be needed to fully characterize the roles of the newly identified locus in neuroprotection from amyloid pathology.

## Conclusion

In this study, we identified a locus on chromosome 3 that modifies the association between baseline CSF amyloid levels and hippocampal atrophy, which our colleagues were able to replicate independently. We also supported previous findings that *APOE*-ɛ4 increases risk for Alzheimer’s disease both upstream and downstream of amyloid pathology. Our results suggest that genes in the axonal branching and synaptic maintenance, along with *APOE*, may be implicated in the downstream consequences of amyloidosis.

## Supplementary Material

fcac066_Supplementary_Data

## Abbreviations

ADNI =: Alzheimer’s Disease Neuroimaging Initiative
AMP-AD =: Accelerating Medicines Partnership Program for Alzheimer’s Disease
APOE =: apolipoprotein E
beta-amyloid =: Aβ, Aβ42
BIOCARD =: Biomarkers of Cognitive Decline Among Normal Individuals
CSF =: cerebrospinal fluid
eQTL =: expression quantitative trait locus
FDR =: false discovery rate
GMM =: Gaussian mixture model
GO =: Gene Ontology
GWAS =: genome-wide association study
GTEx =: NIH Genotype-Tissue Expression Portal
ICV =: intracranial volume
LD =: linkage disequilibrium
MCSA =: Mayo Clinic Study of Aging
mQTL =: methylation quantitative trait locus
MCI =: mild cognitive impairment
MAF =: minor allele frequency
MRI =: magnetic resonance imaging
PheWAS =: phenome-wide association study
PET =: positron emission tomography
PC =: principal component
QC =: quality control
SNP =: single nucleotide polymorphism
SUVR =: standardized uptake value ratio
VMAP =: Vanderbilt Memory and Aging Project
WRAP =: Wisconsin Registry for Alzheimer’s Prevention
