# Supplementary material for: Exploring common genetic contributors to neuroprotection from amyloid pathology
Source: Brain Commun. 2022 Mar 17;4(2):fcac066. doi: 10.1093/braincomms/fcac066 (PMC9006043; doi:10.1093/braincomms/fcac066)
Supplement: fcac066_Supplementary_Data [file fcac066_supplementary_data.pdf]

Participants

The Alzheimer's Disease Neuroimaging Initiative (ADNI) database (adni.loni.usc.edu) was launched in 2003 as a public-private partnership, led by Principal Investigator Michael W. Weiner, MD. The primary goal of ADNI has been to test whether serial MRI, PET, other biological markers, and clinical and neuropsychological assessment can be combined to measure the progression of mild cognitive impairment (MCI) and early AD. ADNI enrolls participants who are 55 to 90 years of age (www.adni-info.org). The Vanderbilt Memory and Aging Project (VMAP) was launched at Vanderbilt University Medical Center. The study enrolls individuals with MCI, as well as age-, sex-, and race-matched cognitively normal counterparts. Participants are excluded if they are not eligible for MRI or have a history of neurological or cardiovascular disease.[1] The Wisconsin Registry for Alzheimer's Prevention (WRAP) began at the University of Wisconsin-Madison in 2001. WRAP enrolls individuals who are cognitively normal, 40 to 65 years of age, have a parental history of AD, and an increased risk for the disease. In 2004, WRAP began recruitment of cognitively normal individuals without a self-reported parental history of AD.[2] The Biomarkers of Cognitive Decline Among Normal Individuals (BIOCARD) study is currently located at Johns Hopkins School of Medicine. BIOCARD enrolls participants who were middle-age and cognitively intact. The study is enriched for individuals who had a first-degree relative with AD.[3]

Data from the Mayo Clinic Study of Aging (MCSA) was used for replication analyses. The MCSA is a population-based prospective study of older adults residing in Olmsted County, Minnesota.[4, 5] Individuals have been enrolled serially starting in 2004, with multimodal clinical and biomarker data obtained at longitudinal visits based on study protocols.

Consent for each participant was acquired by each longitudinal aging study independently. Each study is fully described elsewhere.

Sex and Intracranial Volume Adjustment of Hippocampal Volume

First, all study cohorts were subset to individuals who were cognitively normal at the time of their first MRI scan. Using a linear regression, the slopes (β) of the associations between ICV, sex, and total hippocampal volume and the interaction between ICV and sex (ICV x sex) on total hippocampal volume in cognitively normal individuals, were calculated.

$$Total\ Hippocampal\ Volume \sim ICV + Sex + ICV \times Sex$$

Hippocampal volume for all subjects was then adjusted for the above confounders by subtracting the mean-centered effects of ICV, sex, and ICV x sex from the total hippocampal volume. We performed this adjustment because ICV is associated with hippocampal volume, and ICV also strongly differs by sex [6].

Mayo Clinic Study of Aging Genotype Quality Control

GWAS data for 1783 MCSA participants was acquired using DNA from peripheral blood samples and via the Illumina Infinium Global Screening Array-24 v2.0. Standard SNP-level QC filters were applied using PLINK, including call rate ≥95%, Hardy-Weinberg Equilibrium (p≥1 x 10-5), and MAF ≥1%. Subject-level QC filters included call rate ≥98%, sex checks versus clinical data, Caucasian ancestry determined through STRUCTURE version 2.3.4, and ensuring no cryptic first- or second-degree relatedness (PLINK identity by descent PI_HAT<0.25). Genome-wide imputation was performed with the Michigan Imputation Server using Minimac version 4-1.0.2 and the HRC reference panel. Following additional post-imputation QC filters including SNP call rate ≥95%, sample call rate ≥98%, Hardy-Weinberg Equilibrium (p≥1 x 10-6), MAF≥1%, stringent imputation quality measure (r2≥0.8), and removal of SNPs with duplicate or no identifying rs number, data was available for 6153814 SNPs and 1727 MCSA participants.[7]

MAGMA Gene Sets

Gene sets for MAGMA analyses leveraged data from the Gene Ontology (GO) project,[8] Kyoto Encyclopedia of Genes and Genomes (KEGG),[9-11] REACTOME,[12, 13] BIOCARTA,[14] and the Protein Interaction Database (PID)[15] totaling 12,195 sets.

Supplementary Table 1: Summary of imputation and quality control measures performed on each dataset

		Pre-QC		Pre-imputation		Post-imputation	
Dataset	Platform	Sample size	# of variants	Sample size	# of variants	Sample size	# of variants
ADNI 1	Illumina 610-Quad	757	620,901	666	536,557	666	6,577,704
ADNI 2	Illumina OmniExpress	432	730,525	430	641,075	430	6,662,117
ADNI WGS	Illumina Omni 2.5M (WGS Platform)	812	2,379,855	807	1,480,134	807	7,298,856
ADNI (merged)	(merge)	1903	7,571,217	N/A	N/A	1,247	7,849,615
BIOCARD	Illumina OmniExpress	261	730,525	193	638,868	190	6,559,742
WRAP	Illumina Multi-Ethnic Genotyping Array	1340	1,779,819	1198	898,220	1,198	10,499,994
VMAP	Illumina Infinium Expanded Multi-Ethnic Genotyping Array ($MEGA^{EX}$)	352	1,842,793	261	770,719	256	6,760,400

Note: Since genetic data for ADNI were obtained on multiple platforms, QC and imputation were completed on each set separately and were merged after post-imputation filters. For these merged sets, number of samples and variants immediately after merging and after additional filtering for overlap and relatedness are in the "Pre-QC" and "Post-imputation" columns respectively.

Abbreviations: ADNI=Alzheimer's Disease Neuroimaging Initiative; BIOCARD=Biomarkers of Cognitive Decline Among Normal Individuals cohort; WRAP=Wisconsin Registry for Alzheimer's Prevention; VMAP=Vanderbilt Memory and Aging Project

**Supplementary Table 2:** Summary of protocols for MRI acquisition by site				
Dataset	Scanner Platform	Strength	Image Weight	Protocol
ADNI 1	Multiple (GE, Philips, and Siemens)	1.5 Tesla	T1	www.adni-info.org
ADNI-GO/ADNI-2	Multiple (GE, Philips, and Siemens)	3 Tesla	T1	www.adni-info.org
WRAP	GE MR750	3 Tesla	T1	[16]
VMAP	Philips Achieva	3 Tesla	T1	[1]
BIOCARD	GE	1.5 Tesla	T1	[17]

Abbreviations: ADNI=Alzheimer's Disease Neuroimaging Initiative; BIOCARD=Biomarkers of Cognitive Decline Among Normal Individuals cohort; WRAP=Wisconsin Registry for Alzheimer's Prevention; VMAP=Vanderbilt Memory and Aging Project

Table S3. Suggestive loci for the SNP x CSF AB42 interaction on baseline hippocampal volume; linear mixed-effects models.

SNP	predictor	beta	se	df	t	p
rs62263260_T	rs62263260_T:interval:std_mixture_ab42	0.03302265	0.00595026	2809	5.5497793	3.12717E-08
rs11707826_T	rs11707826_T:interval:std_mixture_ab42	0.03295566	0.00594666	2809	5.54187714	3.27005E-08
rs10934626_T	rs10934626_T:interval:std_mixture_ab42	0.03295566	0.00594666	2809	5.54187714	3.27005E-08
rs4677869_T	rs4677869_T:interval:std_mixture_ab42	0.03182185	0.00594324	2809	5.35429276	9.2828E-08
rs2288678_G	rs2288678_G:interval:std_mixture_ab42	0.03195757	0.00621294	2809	5.14370772	2.879E-07
rs11706210_C	rs11706210_C:interval:std_mixture_ab42	0.03103419	0.00620389	2809	5.00237522	6.0116E-07
rs11707039_A	rs11707039_A:interval:std_mixture_ab42	0.03103419	0.00620389	2809	5.00237522	6.0116E-07
rs938591_C	rs938591_C:interval:std_mixture_ab42	0.02329748	0.00467336	2809	4.98516511	6.56703E-07
rs10833448_T	rs10833448_T:interval:std_mixture_ab42	0.02325151	0.00467397	2809	4.97468278	6.92924E-07
rs4677982_C	rs4677982_C:interval:std_mixture_ab42	0.03066945	0.00617275	2809	4.96852271	7.151E-07
rs4677983_A	rs4677983_A:interval:std_mixture_ab42	0.03050867	0.00617145	2809	4.94351384	8.12364E-07
rs4677984_T	rs4677984_T:interval:std_mixture_ab42	0.03050867	0.00617145	2809	4.94351384	8.12364E-07
rs4677870_C	rs4677870_C:interval:std_mixture_ab42	0.03050867	0.00617145	2809	4.94351384	8.12364E-07
rs10934625_C	rs10934625_C:interval:std_mixture_ab42	0.03044451	0.00617403	2809	4.93105591	8.65455E-07
rs35989119_C	rs35989119_C:interval:std_mixture_ab42	0.03043541	0.00617418	2809	4.92946592	8.72467E-07
rs4536170_C	rs4536170_C:interval:std_mixture_ab42	0.02118637	0.00466847	2809	4.53818205	5.91252E-06
rs1037731_G	rs1037731_G:interval:std_mixture_ab42	0.02106634	0.00466543	2809	4.51540906	6.57982E-06
rs963960_T	rs963960_T:interval:std_mixture_ab42	0.02106634	0.00466543	2809	4.51540906	6.57982E-06
rs4542368_T	rs4542368_T:interval:std_mixture_ab42	0.02097454	0.00466555	2809	4.49561884	7.21773E-06
rs12276382_C	rs12276382_C:interval:std_mixture_ab42	0.02083416	0.00470447	2809	4.42858321	9.84778E-06
rs7935097_T	rs7935097_T:interval:std_mixture_ab42	0.02040312	0.00471351	2809	4.32864396	1.55276E-05
rs4753405_T	rs4753405_T:interval:std_mixture_ab42	0.02040312	0.00471351	2809	4.32864396	1.55276E-05
rs116647170_T	rs116647170_T:interval:std_mixture_ab42	0.1071517	0.02478086	2809	4.32396988	1.58583E-05
rs6483171_A	rs6483171_A:interval:std_mixture_ab42	0.02035471	0.00470999	2809	4.32160241	1.60283E-05
rs7944251_A	rs7944251_A:interval:std_mixture_ab42	0.02035471	0.00470999	2809	4.32160241	1.60283E-05
rs12576325_T	rs12576325_T:interval:std_mixture_ab42	0.02016764	0.00471459	2809	4.2777051	1.95137E-05
rs12786200_T	rs12786200_T:interval:std_mixture_ab42	0.02016764	0.00471459	2809	4.2777051	1.95137E-05

rs2212387_T	rs2212387_T:interval:std_mixture_ab42	0.02016764	0.00471459	2809	4.2777051	1.95137E-05
rs7934732_T	rs7934732_T:interval:std_mixture_ab42	0.02016764	0.00471459	2809	4.2777051	1.95137E-05
rs2054215_A	rs2054215_A:interval:std_mixture_ab42	0.02009833	0.00471186	2809	4.26548089	2.06062E-05
rs77107095_A	rs77107095_A:interval:std_mixture_ab42	-0.0424072	0.0099428	2809	-4.2651125	2.064E-05
rs112636864_A	rs112636864_A:interval:std_mixture_ab42	-0.0424072	0.0099428	2809	-4.2651125	2.064E-05
rs113395363_T	rs113395363_T:interval:std_mixture_ab42	-0.0424072	0.0099428	2809	-4.2651125	2.064E-05
rs74822708_A	rs74822708_A:interval:std_mixture_ab42	-0.0424072	0.0099428	2809	-4.2651125	2.064E-05
rs11120645_T	rs11120645_T:interval:std_mixture_ab42	-0.0424072	0.0099428	2809	-4.2651125	2.064E-05
rs113393658_A	rs113393658_A:interval:std_mixture_ab42	-0.0426389	0.01001382	2809	-4.2580066	2.13026E-05
rs146752822_A	rs146752822_A:interval:std_mixture_ab42	-0.0426389	0.01001382	2809	-4.2580066	2.13026E-05
rs12132798_G	rs12132798_G:interval:std_mixture_ab42	-0.0426389	0.01001382	2809	-4.2580066	2.13026E-05
rs12577025_A	rs12577025_A:interval:std_mixture_ab42	0.02004557	0.00471111	2809	4.25495344	2.15935E-05
rs10501774_A	rs10501774_A:interval:std_mixture_ab42	0.02004557	0.00471111	2809	4.25495344	2.15935E-05
rs1320636_A	rs1320636_A:interval:std_mixture_ab42	0.02004557	0.00471111	2809	4.25495344	2.15935E-05
rs12144789_T	rs12144789_T:interval:std_mixture_ab42	-0.0422474	0.0099379	2809	-4.2511337	2.19627E-05
rs12123296_T	rs12123296_T:interval:std_mixture_ab42	-0.0423437	0.01001321	2809	-4.2287851	2.42464E-05
rs12126534_A	rs12126534_A:interval:std_mixture_ab42	-0.0423437	0.01001321	2809	-4.2287851	2.42464E-05
rs112735413_A	rs112735413_A:interval:std_mixture_ab42	-0.0423437	0.01001321	2809	-4.2287851	2.42464E-05
rs75410548_C	rs75410548_C:interval:std_mixture_ab42	-0.0423437	0.01001321	2809	-4.2287851	2.42464E-05
rs76992333_T	rs76992333_T:interval:std_mixture_ab42	-0.0423437	0.01001321	2809	-4.2287851	2.42464E-05
rs16917214_T	rs16917214_T:interval:std_mixture_ab42	0.01988752	0.00471024	2809	4.2221912	2.49623E-05
rs16917218_A	rs16917218_A:interval:std_mixture_ab42	0.01988752	0.00471024	2809	4.2221912	2.49623E-05
rs74592637_A	rs74592637_A:interval:std_mixture_ab42	-0.0566469	0.01346481	2809	-4.2070333	2.6685E-05
rs2371519_G	rs2371519_G:interval:std_mixture_ab42	-0.0566469	0.01346481	2809	-4.2070333	2.6685E-05
rs11108857_C	rs11108857_C:interval:std_mixture_ab42	-0.0566469	0.01346481	2809	-4.2070333	2.6685E-05
rs4314861_G	rs4314861_G:interval:std_mixture_ab42	-0.0414655	0.00992167	2809	-4.1792829	3.01359E-05
rs7416880_A	rs7416880_A:interval:std_mixture_ab42	-0.0414655	0.00992167	2809	-4.1792829	3.01359E-05
rs12129576_C	rs12129576_C:interval:std_mixture_ab42	-0.0414655	0.00992167	2809	-4.1792829	3.01359E-05
rs12132110_A	rs12132110_A:interval:std_mixture_ab42	-0.0414655	0.00992167	2809	-4.1792829	3.01359E-05
rs74447991_T	rs74447991_T:interval:std_mixture_ab42	-0.0414655	0.00992167	2809	-4.1792829	3.01359E-05
rs12140781_A	rs12140781_A:interval:std_mixture_ab42	-0.0414655	0.00992167	2809	-4.1792829	3.01359E-05
rs12137445_A	rs12137445_A:interval:std_mixture_ab42	-0.0414297	0.00992011	2809	-4.176332	3.05269E-05
rs12139361_A	rs12139361_A:interval:std_mixture_ab42	-0.0414297	0.00992011	2809	-4.176332	3.05269E-05
rs4453013_A	rs4453013_A:interval:std_mixture_ab42	-0.0414297	0.00992011	2809	-4.176332	3.05269E-05

rs12138094_A	rs12138094_A:interval:std_mixture_ab42	-0.0414297	0.00992011	2809	-4.176332	3.05269E-05
rs79937377_A	rs79937377_A:interval:std_mixture_ab42	-0.0414297	0.00992011	2809	-4.176332	3.05269E-05
rs12141340_C	rs12141340_C:interval:std_mixture_ab42	-0.0414297	0.00992011	2809	-4.176332	3.05269E-05
rs11120650_T	rs11120650_T:interval:std_mixture_ab42	-0.0414297	0.00992011	2809	-4.176332	3.05269E-05
rs1416842_A	rs1416842_A:interval:std_mixture_ab42	-0.0416625	0.00999137	2809	-4.1698475	3.1403E-05
rs79789651_C	rs79789651_C:interval:std_mixture_ab42	-0.0416625	0.00999137	2809	-4.1698475	3.1403E-05
rs12117370_A	rs12117370_A:interval:std_mixture_ab42	-0.0416576	0.00999132	2809	-4.1693784	3.14673E-05
rs12409223_G	rs12409223_G:interval:std_mixture_ab42	-0.0416576	0.00999132	2809	-4.1693784	3.14673E-05
rs112141149_G	rs112141149_G:interval:std_mixture_ab42	-0.0416576	0.00999132	2809	-4.1693784	3.14673E-05
rs12132976_A	rs12132976_A:interval:std_mixture_ab42	-0.0412577	0.00991514	2809	-4.1610836	3.26252E-05
rs12135237_A	rs12135237_A:interval:std_mixture_ab42	-0.0412577	0.00991514	2809	-4.1610836	3.26252E-05
rs12133551_C	rs12133551_C:interval:std_mixture_ab42	-0.0412577	0.00991514	2809	-4.1610836	3.26252E-05
rs4651332_T	rs4651332_T:interval:std_mixture_ab42	0.02111807	0.00508223	2809	4.15527614	3.34598E-05
rs41277194_C	rs41277194_C:interval:std_mixture_ab42	-0.0413826	0.00999094	2809	-4.1420168	3.54421E-05
rs12402107_G	rs12402107_G:interval:std_mixture_ab42	-0.0413826	0.00999094	2809	-4.1420168	3.54421E-05
rs76191410_A	rs76191410_A:interval:std_mixture_ab42	-0.0413826	0.00999094	2809	-4.1420168	3.54421E-05
rs12126346_C	rs12126346_C:interval:std_mixture_ab42	-0.0413826	0.00999094	2809	-4.1420168	3.54421E-05
rs1934430_T	rs1934430_T:interval:std_mixture_ab42	-0.0413779	0.00999086	2809	-4.1415744	3.55102E-05
rs7521601_T	rs7521601_T:interval:std_mixture_ab42	0.02085043	0.00512864	2809	4.06548853	4.92483E-05
rs7532616_A	rs7532616_A:interval:std_mixture_ab42	0.02085043	0.00512864	2809	4.06548853	4.92483E-05
rs6425052_C	rs6425052_C:interval:std_mixture_ab42	0.02085043	0.00512864	2809	4.06548853	4.92483E-05
rs6687896_G	rs6687896_G:interval:std_mixture_ab42	0.02085043	0.00512864	2809	4.06548853	4.92483E-05
rs7541946_C	rs7541946_C:interval:std_mixture_ab42	0.02085043	0.00512864	2809	4.06548853	4.92483E-05
rs12137796_T	rs12137796_T:interval:std_mixture_ab42	-0.0416803	0.01026162	2809	-4.0617644	5.0036E-05
rs141173782_T	rs141173782_T:interval:std_mixture_ab42	-0.0416803	0.01026162	2809	-4.0617644	5.0036E-05
rs12406554_T	rs12406554_T:interval:std_mixture_ab42	-0.0416803	0.01026162	2809	-4.0617644	5.0036E-05
rs12129954_C	rs12129954_C:interval:std_mixture_ab42	-0.0416803	0.01026162	2809	-4.0617644	5.0036E-05
rs4651334_A	rs4651334_A:interval:std_mixture_ab42	0.02060012	0.00507335	2809	4.06045875	5.0315E-05
rs7196791_T	rs7196791_T:interval:std_mixture_ab42	-0.0244444	0.00608769	2809	-4.015383	6.09036E-05
rs10732978_A	rs10732978_A:interval:std_mixture_ab42	0.02052538	0.00512647	2809	4.0038055	6.3946E-05
rs6688584_T	rs6688584_T:interval:std_mixture_ab42	0.02052538	0.00512647	2809	4.0038055	6.3946E-05
rs10911934_A	rs10911934_A:interval:std_mixture_ab42	0.02052538	0.00512647	2809	4.0038055	6.3946E-05
rs10737270_T	rs10737270_T:interval:std_mixture_ab42	0.02052538	0.00512647	2809	4.0038055	6.3946E-05
rs10798060_C	rs10798060_C:interval:std_mixture_ab42	0.02052538	0.00512647	2809	4.0038055	6.3946E-05

rs6685652_C	rs6685652_C:interval:std_mixture_ab42	0.02052538	0.00512647	2809	4.0038055	6.3946E-05
rs10752977_G	rs10752977_G:interval:std_mixture_ab42	0.02052538	0.00512647	2809	4.0038055	6.3946E-05
rs10752978_A	rs10752978_A:interval:std_mixture_ab42	0.02052538	0.00512647	2809	4.0038055	6.3946E-05
rs10752979_A	rs10752979_A:interval:std_mixture_ab42	0.02052538	0.00512647	2809	4.0038055	6.3946E-05
rs10798061_A	rs10798061_A:interval:std_mixture_ab42	0.02052538	0.00512647	2809	4.0038055	6.3946E-05
rs10911933_C	rs10911933_C:interval:std_mixture_ab42	0.0205026	0.00512904	2809	3.99735638	6.57027E-05
rs12141208_C	rs12141208_C:interval:std_mixture_ab42	-0.0406683	0.01023884	2809	-3.9719604	7.30743E-05
rs113042941_C	rs113042941_C:interval:std_mixture_ab42	-0.0406683	0.01023884	2809	-3.9719604	7.30743E-05
rs79825305_A	rs79825305_A:interval:std_mixture_ab42	-0.0406627	0.01023884	2809	-3.9714151	7.32409E-05
rs75574076_T	rs75574076_T:interval:std_mixture_ab42	-0.0406473	0.01023685	2809	-3.9706838	7.34648E-05
rs112167269_G	rs112167269_G:interval:std_mixture_ab42	-0.0402616	0.01015192	2809	-3.9659125	7.49418E-05
rs12130528_C	rs12130528_C:interval:std_mixture_ab42	-0.0402616	0.01015192	2809	-3.9659125	7.49418E-05
rs6425053_A	rs6425053_A:interval:std_mixture_ab42	0.02022138	0.00518655	2809	3.89881194	9.89279E-05
rs2205897_T	rs2205897_T:interval:std_mixture_ab42	0.01972771	0.0050669	2809	3.89344457	0.000101131
rs10489407_A	rs10489407_A:interval:std_mixture_ab42	0.01966908	0.00506511	2809	3.88325252	0.000105444
rs4130590_A	rs4130590_A:interval:std_mixture_ab42	-0.0143884	0.00389459	2809	-3.6944712	0.000224548
rs7041940_T	rs7041940_T:interval:std_mixture_ab42	-0.0143884	0.00389459	2809	-3.6944712	0.000224548
rs6478770_T	rs6478770_T:interval:std_mixture_ab42	-0.0142242	0.0038977	2809	-3.6493869	0.000267654
rs10279772_A	rs10279772_A:interval:std_mixture_ab42	-0.0163002	0.00451725	2809	-3.6084391	0.000313421
rs10280089_A	rs10280089_A:interval:std_mixture_ab42	-0.0163002	0.00451725	2809	-3.6084391	0.000313421
rs1363073_T	rs1363073_T:interval:std_mixture_ab42	0.01740418	0.00499703	2809	3.4829033	0.000503585
rs3096644_T	rs3096644_T:interval:std_mixture_ab42	-0.0150429	0.00437935	2809	-3.4349683	0.000601218
rs3110609_C	rs3110609_C:interval:std_mixture_ab42	-0.0148135	0.0044032	2809	-3.3642604	0.00077779

**Supplementary Table 4:** rs62263260 x β-Amyloid results stratified by diagnosis and aging study					
**Sample stratified by diagnosis**					
Sample	N	% rs62263260 minor allele (T) carriers	B	SE	P value
Normal Cognition	490	19.8	0.0127	0.0037	0.0006
MCI	475	22.9	0.0174	0.0046	0.0002
AD	100	28.0	0.0188	0.0134	0.1657
**Sample stratified by aging study**					
Sample	N	% rs62263260 minor allele (T) carriers	B	SE	P value
ADNI	702	22.5	0.0102	0.0036	0.0041
VMAP	126	20.6	0.0322	0.0069	8.07e-6
WRAP	97	24.7	0.0053	0.0150	0.722
BIOCARD	140	18.6	0.0119	0.0121	0.328

**Supplementary Table 5:** All coefficients from rs62263260 x β-Amyloid model when additional age x dataset covariate			
Model: Annual change in HV ~ age at baseline MRI + sex + PC1-3 + rs62263260*CSF Aβ42 + age at baseline MRI x dataset			
Predictor	B	SE	P value
Intercept	0.0128	0.03	0.67
Sex	-0.0019	0.005	0.673
Age at baseline MRI	-0.002	0.0004	7.88e-7
PC1	0.0418	0.113	0.711
PC2	-0.023	0.127	0.856
PC3	-0.057	0.109	0.604
rs62263260	0.0147	0.006	0.015
CSF Aβ42	0.013	0.002	< 2.00e-16
BIOCARD	-0.136	0.057	0.016
VMAP	0.114	0.0797	0.151
WRAP	0.069	0.085	0.413
rs62263260 x CSF Aβ42	0.0151	0.003	7.32e-8
Age x BIOCARD	0.0025	0.0009	0.0045
Age x VMAP	-0.0015	0.001	0.168
Age x WRAP	-0.0002	0.001	0.889

Supplementary Table 6: Dataset characteristics by analysis (CSF and PET)

	Combined CSF Dataset[a]	CSF GWAS Dataset	ADNI PET Total
N	1537	1065	667
Age at baseline	70.3 ± 8.8	70.8±8.7	73.4±7.1
Sex, % female	48%	47%	44%
% *APOE*-ε4 carriers	42%	40%	41%
% *APOE*-ε2 carriers	10%	10%	10%
NC (persons)	734	490	220
MCI (persons)	637	475	398
AD (persons)	176	100	49
Std. CSF Aβ42	-1.31±1.7	-1.34±1.7	2.53±3.07
Neuroimaging Measurements (MRI)			
Std. Hippocampal Volume	-0.55±1.3	-0.58±1.3	-0.62±1.3
Std. Hippocampal Vol. Slopes	-0.13±0.1	-0.14±0.1	-0.16±0.1

Table S7. Coloc results for genes within the 1Mb region of rs62263260; only SEMA5B were tested in GTEx tissues because rs62263260 (or its associated SNPs) was not an eQTL for any other gene in GTEx. In GTEx significance is p<0.0011

Sieberts et al., 2020; AMP-AD Cortex Meta-Analysis eQTL, n=1433							
**Gene**	**SNP**	**eQTL pvalue (FDR-corrected)**	**PP3**	**PP4**	**Best-fit SNP**	**BP Position (hg38)**	**Best-fit SNP.PP.H4**
SEMA5B	rs62263260	9.31E-07	0.9980	6.33E-05	rs2276782	3:122913049	0.8688
CCDC58	rs62263260	0.7853	0.346	0.0338	rs62263260	3:122956480	0.2711
FAM162A	rs62263260	0.4372	0.175	0.106	rs10934626	3:122957676	0.3026
WDR5B	rs62263260	0.3234	0.9980	8.93E-05	rs6438741	3:122562494	0.2564
KPNA1	rs62263260	0.9854	0.9980	3.32E-05	rs6771095	3:122472888	0.1279
PARP9	rs62263260	0.9930	0.9980	5.51E-05	rs9872708	3:122523960	0.0570
DTX3L	rs62263260	0.8757	0.136	0.0347	rs62263260	3:122956480	0.2790
PARP14	rs62263260	0.9436	0.9980	6.01E-05	rs10804558	3:122826566	0.2284
HSPBAP1	rs62263260	0.7977	0.9980	5.83E-05	rs2276775	3:122927887	0.9914
DIRC2	rs62263260	0.0680	0.9960	0.0013	rs2276781	3:122913589	0.1711
PDIA5	rs62263260	0.8578	0.449	0.0247	rs62263260	3:122956480	0.2599
SEC22A	rs62263260	0.5753	0.9970	1.31E-04	rs62263260	3:122956480	0.1934
ADCY5	rs62263260	0.9027	0.187	0.0484	rs10934626	3:122957676	0.1830
HACD2	rs62263260	0.8443	0.205	0.0353	rs62263260	3:122956480	0.2718
MYLK	rs62263260	0.9457	0.139	0.0334	rs62263260	3:122956480	0.2482
CCDC14	rs62263260	0.8065	0.278	0.0363	rs62263260	3:122956480	0.2636

GTEx v8 - Esophagus Muscularis, n=465							
**Gene**	**SNP**	**eQTL p-value (uncorrected)**	**PP3**	**PP4**	**Best-fit SNP**	**BP Position (hg38)**	**Best-fit SNP.PP.H4**
SEMA5B	rs62263260	0.0000019	0.0020	0.9950	rs10934626	3:122957676	0.4187

GTEx v8 - Testis, n=368							
**Gene**	**SNP**	**eQTL p-value (uncorrected)**	**PP3**	**PP4**	**Best-fit SNP**	**BP Position (hg38)**	**Best-fit SNP.PP.H4**
SEMA5B	rs10934626	0.000095	0.9890	0.0064	rs10934626	3:122957676	0.4900

GTEx v8 -Brain Anterior Cingulate Cortex BA24, n=147

Gene	SNP	eQTL p-value (uncorrected)	PP3	PP4	Best-fit SNP	BP Position (hg38)	Best-fit SNP.PP.H4
SEMA5B	rs62263260	0.00048	9.39E-04	0.0267	rs16833588	3:122857345	1.20E-05

**Supplementary Table 8:** Breakdown of combined GWAS dataset by cohort						
	ADNI	VMAP	BIOCARD	WRAP	Total	P value
N	702	126	140	97	1065	<0.001
%Female	44%	31%	61%	65%	47%	<0.001
Age, years	73.6±7.1	73.0±6.4	60.8±7.7	61.7±5.7	70.8±8.7	<0.001
%APOE-ε4 Carriers	43%	33%	35%	38%	40%	0.059
%APOE-ε2 Carriers	10%	10%	11%	14%	10%	0.474
Standardized Aβ Value	-1.55±1.6	-1.73±2.0	-0.45±1.4	-0.55±1.4	-1.34±1.7	<0.001
Hippocampal Volume	-0.81±1.4	-0.43±1.3	0.001±1.0	0.09±1.0	-0.58±1.3	<0.001
Hippocampal Slopes	-0.15±0.8	-0.15±0.1	-0.09±0.1	-0.06±0.1	-0.14±0.1	<0.001
Number of Visits	4.04±1.94	2.97±0.90	2.29±1.35	3.59±1.26	3.64±1.83	<0.001

Table S9. Suggestive loci for the SNP x CSF AB42 interaction on baseline hippocampal volume.

CHR	SNP	BP	A1	TEST	N	Beta	SE	L95	U95	T-STAT	P
2	rs72792463	29661158	G	ADDxstd_mixture_ab42	1065	-0.1942	0.04373	-0.28	-0.1085	-4.442	9.86E-06
2	rs55902725	71694837	G	ADDxstd_mixture_ab42	1065	0.2373	0.05196	0.1354	0.3391	4.566	5.54E-06
3	rs814752	65783033	T	ADDxstd_mixture_ab42	1065	-0.1669	0.03652	-0.2385	-0.09536	-4.571	5.43E-06
5	rs168660	154715368	G	ADDxstd_mixture_ab42	1065	0.1659	0.03687	0.0936	0.2381	4.499	7.59E-06
5	rs1820796	154734068	C	ADDxstd_mixture_ab42	1065	0.1659	0.03687	0.0936	0.2381	4.499	7.59E-06
5	rs1564800	154761128	A	ADDxstd_mixture_ab42	1065	0.1775	0.03717	0.1046	0.2504	4.775	2.05E-06
6	rs77207148	139949556	A	ADDxstd_mixture_ab42	1065	-0.3101	0.06289	-0.4333	-0.1868	-4.931	9.52E-07
6	rs74419807	139950129	C	ADDxstd_mixture_ab42	1065	-0.3101	0.06289	-0.4333	-0.1868	-4.931	9.52E-07
6	rs79140486	139952778	A	ADDxstd_mixture_ab42	1065	-0.3101	0.06289	-0.4333	-0.1868	-4.931	9.52E-07
6	rs2185921	139955081	A	ADDxstd_mixture_ab42	1065	-0.3096	0.06242	-0.432	-0.1873	-4.96	8.21E-07
6	rs116083533	139956288	A	ADDxstd_mixture_ab42	1065	-0.2904	0.06259	-0.413	-0.1677	-4.639	3.94E-06
8	rs1527033	24889912	T	ADDxstd_mixture_ab42	1065	0.1444	0.03157	0.08256	0.2063	4.575	5.32E-06
8	rs2320780	24890137	C	ADDxstd_mixture_ab42	1065	0.1434	0.03159	0.08149	0.2053	4.539	6.29E-06
8	rs4872283	24892440	G	ADDxstd_mixture_ab42	1065	0.1436	0.03154	0.08176	0.2054	4.552	5.94E-06
8	rs4425762	24892833	C	ADDxstd_mixture_ab42	1065	0.1436	0.03154	0.08176	0.2054	4.552	5.94E-06
8	rs2320782	24915220	T	ADDxstd_mixture_ab42	1065	0.1455	0.03146	0.08381	0.2071	4.624	4.23E-06
8	rs11135840	24924178	A	ADDxstd_mixture_ab42	1065	0.1549	0.03111	0.09396	0.2159	4.98	7.42E-07
8	rs7017203	24925058	C	ADDxstd_mixture_ab42	1065	0.1549	0.03111	0.09396	0.2159	4.98	7.42E-07
8	rs9314297	24925366	G	ADDxstd_mixture_ab42	1065	0.1539	0.03114	0.09289	0.215	4.943	8.96E-07
9	rs404479	23479588	T	ADDxstd_mixture_ab42	1065	0.1352	0.02943	0.0775	0.1929	4.593	4.89E-06
17	rs986993	26187576	A	ADDxstd_mixture_ab42	1065	-0.1514	0.034	-0.218	-0.08476	-4.453	9.35E-06
17	rs7220473	26194116	T	ADDxstd_mixture_ab42	1065	-0.1558	0.03383	-0.2221	-0.08953	-4.606	4.60E-06
19	rs77105559	15687082	A	ADDxstd_mixture_ab42	1065	-0.3966	0.08879	-0.5706	-0.2226	-4.467	8.78E-06
19	rs114237593	15687203	C	ADDxstd_mixture_ab42	1065	-0.3966	0.08879	-0.5706	-0.2226	-4.467	8.78E-06
19	rs77287595	15688045	T	ADDxstd_mixture_ab42	1065	-0.3966	0.08879	-0.5706	-0.2226	-4.467	8.78E-06
19	rs74978423	15693290	C	ADDxstd_mixture_ab42	1065	-0.3989	0.0884	-0.5722	-0.2257	-4.513	7.13E-06
19	rs74701558	15693707	G	ADDxstd_mixture_ab42	1065	-0.3989	0.0884	-0.5722	-0.2257	-4.513	7.13E-06
19	rs115557777	15693920	T	ADDxstd_mixture_ab42	1065	-0.3989	0.0884	-0.5722	-0.2257	-4.513	7.13E-06
19	rs16980583	15695122	A	ADDxstd_mixture_ab42	1065	-0.3989	0.0884	-0.5722	-0.2257	-4.513	7.13E-06
19	rs114636154	15695242	A	ADDxstd_mixture_ab42	1065	-0.3989	0.0884	-0.5722	-0.2257	-4.513	7.13E-06
19	rs148622580	15696277	T	ADDxstd_mixture_ab42	1065	-0.3989	0.0884	-0.5722	-0.2257	-4.513	7.13E-06
19	rs16980587	15697034	C	ADDxstd_mixture_ab42	1065	-0.3989	0.0884	-0.5722	-0.2257	-4.513	7.13E-06
19	rs283815	45390333	G	ADDxstd_mixture_ab42	1065	0.157	0.03382	0.09068	0.2233	4.641	3.90E-06
19	rs184017	45394969	G	ADDxstd_mixture_ab42	1065	0.1536	0.0338	0.08737	0.2199	4.545	6.14E-06
19	rs157581	45395714	C	ADDxstd_mixture_ab42	1065	0.1535	0.0338	0.08723	0.2197	4.541	6.25E-06
19	rs157582	45396219	T	ADDxstd_mixture_ab42	1065	0.155	0.03382	0.08867	0.2212	4.582	5.16E-06
20	rs1884197	12872365	T	ADDxstd_mixture_ab42	1065	-0.1924	0.041	-0.2728	-0.112	-4.692	3.06E-06
20	rs2423712	12875977	T	ADDxstd_mixture_ab42	1065	-0.1871	0.04102	-0.2675	-0.1067	-4.562	5.66E-06
20	rs6033541	12880005	A	ADDxstd_mixture_ab42	1065	-0.1811	0.04071	-0.2608	-0.1013	-4.447	9.63E-06
20	rs926902	12880880	T	ADDxstd_mixture_ab42	1065	-0.1809	0.04072	-0.2607	-0.1011	-4.442	9.85E-06
20	rs1321951	12882115	C	ADDxstd_mixture_ab42	1065	-0.1873	0.04097	-0.2676	-0.107	-4.573	5.38E-06
20	rs768875	12882649	G	ADDxstd_mixture_ab42	1065	-0.2005	0.04102	-0.2809	-0.1201	-4.888	1.18E-06
20	rs1624243	12884535	T	ADDxstd_mixture_ab42	1065	-0.2005	0.04102	-0.2809	-0.1201	-4.888	1.18E-06
20	rs562596	12885700	C	ADDxstd_mixture_ab42	1065	-0.2005	0.04102	-0.2809	-0.1201	-4.888	1.18E-06
20	rs845710	12887609	G	ADDxstd_mixture_ab42	1065	-0.2005	0.04102	-0.2809	-0.1201	-4.888	1.18E-06
20	rs439580	12888105	C	ADDxstd_mixture_ab42	1065	-0.2005	0.04102	-0.2809	-0.1201	-4.888	1.18E-06
20	rs550630	12890927	T	ADDxstd_mixture_ab42	1065	-0.1992	0.04102	-0.2796	-0.1188	-4.856	1.38E-06
20	rs552504	12891137	T	ADDxstd_mixture_ab42	1065	-0.1988	0.04102	-0.2792	-0.1184	-4.845	1.45E-06

Table S10. Suggestive loci for the SNP x CSF AB42 interaction on the annual change in hippocampal volume. Highlighted SNPs were included as main results in the manuscript.

CHR	SNP	BP	A1	TEST	N	Beta	SE	L95	U95	T-STAT	P
1	rs2205897	186812367	T	ADDxstd_mixture_ab42	1065	0.01105	0.00246	0.006231	0.01588	4.493	7.81E-06
1	rs10489407	186824113	A	ADDxstd_mixture_ab42	1065	0.01091	0.002457	0.006095	0.01573	4.44	9.93E-06
1	rs4651332	186826361	T	ADDxstd_mixture_ab42	1065	0.01143	0.002468	0.006593	0.01627	4.631	4.09E-06
1	rs4651334	186826844	A	ADDxstd_mixture_ab42	1065	0.01124	0.002464	0.006413	0.01607	4.563	5.64E-06
1	rs10732978	186827843	A	ADDxstd_mixture_ab42	1065	0.01143	0.002488	0.006551	0.0163	4.593	4.89E-06
1	rs6688584	186829074	T	ADDxstd_mixture_ab42	1065	0.01143	0.002488	0.006551	0.0163	4.593	4.89E-06
1	rs10911933	186830564	C	ADDxstd_mixture_ab42	1065	0.01142	0.002489	0.006546	0.0163	4.59	4.97E-06
1	rs10911934	186830636	A	ADDxstd_mixture_ab42	1065	0.01143	0.002488	0.006551	0.0163	4.593	4.89E-06
1	rs10737270	186831073	T	ADDxstd_mixture_ab42	1065	0.01143	0.002488	0.006551	0.0163	4.593	4.89E-06
1	rs10798060	186831513	C	ADDxstd_mixture_ab42	1065	0.01143	0.002488	0.006551	0.0163	4.593	4.89E-06
1	rs6685652	186831728	C	ADDxstd_mixture_ab42	1065	0.01143	0.002488	0.006551	0.0163	4.593	4.89E-06
1	rs10752977	186834039	G	ADDxstd_mixture_ab42	1065	0.01143	0.002488	0.006551	0.0163	4.593	4.89E-06
1	rs10752978	186835740	A	ADDxstd_mixture_ab42	1065	0.01143	0.002488	0.006551	0.0163	4.593	4.89E-06
1	rs10752979	186835995	A	ADDxstd_mixture_ab42	1065	0.01143	0.002488	0.006551	0.0163	4.593	4.89E-06
1	rs10798061	186836066	A	ADDxstd_mixture_ab42	1065	0.01143	0.002488	0.006551	0.0163	4.593	4.89E-06
1	rs7521601	186836394	T	ADDxstd_mixture_ab42	1065	0.01154	0.002489	0.006661	0.01642	4.636	3.99E-06
1	rs7532616	186836889	A	ADDxstd_mixture_ab42	1065	0.01154	0.002489	0.006661	0.01642	4.636	3.99E-06
1	rs6425052	186837191	C	ADDxstd_mixture_ab42	1065	0.01154	0.002489	0.006661	0.01642	4.636	3.99E-06
1	rs6687896	186837421	G	ADDxstd_mixture_ab42	1065	0.01154	0.002489	0.006661	0.01642	4.636	3.99E-06
1	rs6425053	186837754	A	ADDxstd_mixture_ab42	1065	0.01127	0.002512	0.006351	0.0162	4.488	7.96E-06
1	rs7541946	186838268	C	ADDxstd_mixture_ab42	1065	0.01154	0.002489	0.006661	0.01642	4.636	3.99E-06
1	rs75574076	215969990	T	ADDxstd_mixture_ab42	1065	-0.02433	0.005185	-0.03449	-0.01416	-4.691	3.07E-06
1	rs4314861	215970771	G	ADDxstd_mixture_ab42	1065	-0.02438	0.00503	-0.03423	-0.01452	-4.846	1.45E-06
1	rs7416880	215971402	A	ADDxstd_mixture_ab42	1065	-0.02438	0.00503	-0.03423	-0.01452	-4.846	1.45E-06
1	rs12129576	215972121	C	ADDxstd_mixture_ab42	1065	-0.02438	0.00503	-0.03423	-0.01452	-4.846	1.45E-06
1	rs12132110	215972180	A	ADDxstd_mixture_ab42	1065	-0.02438	0.00503	-0.03423	-0.01452	-4.846	1.45E-06
1	rs74447991	215972980	T	ADDxstd_mixture_ab42	1065	-0.02438	0.00503	-0.03423	-0.01452	-4.846	1.45E-06
1	rs12140781	215973131	A	ADDxstd_mixture_ab42	1065	-0.02438	0.00503	-0.03423	-0.01452	-4.846	1.45E-06
1	rs112167269	215974593	G	ADDxstd_mixture_ab42	1065	-0.02402	0.005139	-0.03409	-0.01395	-4.674	3.34E-06
1	rs12132976	215978592	A	ADDxstd_mixture_ab42	1065	-0.02411	0.005026	-0.03396	-0.01426	-4.797	1.84E-06
1	rs12130528	215978891	C	ADDxstd_mixture_ab42	1065	-0.02402	0.005139	-0.03409	-0.01395	-4.674	3.34E-06

1	rs12135237	215981038	A	ADDxstd_mixture_ab42	1065	-0.02411	0.005026	-0.03396	-0.01426	-4.797	1.84E-06
1	rs12133551	215981542	C	ADDxstd_mixture_ab42	1065	-0.02411	0.005026	-0.03396	-0.01426	-4.797	1.84E-06
1	rs12144789	215982435	T	ADDxstd_mixture_ab42	1065	-0.0247	0.005039	-0.03458	-0.01483	-4.903	1.09E-06
1	rs77107095	215984842	A	ADDxstd_mixture_ab42	1065	-0.02476	0.00504	-0.03464	-0.01488	-4.913	1.04E-06
1	rs112636864	215985470	A	ADDxstd_mixture_ab42	1065	-0.02476	0.00504	-0.03464	-0.01488	-4.913	1.04E-06
1	rs113395363	215985617	T	ADDxstd_mixture_ab42	1065	-0.02476	0.00504	-0.03464	-0.01488	-4.913	1.04E-06
1	rs12137796	215988310	T	ADDxstd_mixture_ab42	1065	-0.02512	0.005219	-0.03535	-0.01489	-4.814	1.70E-06
1	rs74822708	215988582	A	ADDxstd_mixture_ab42	1065	-0.02476	0.00504	-0.03464	-0.01488	-4.913	1.04E-06
1	rs141173782	215988815	T	ADDxstd_mixture_ab42	1065	-0.02512	0.005219	-0.03535	-0.01489	-4.814	1.70E-06
1	rs11120645	215990479	T	ADDxstd_mixture_ab42	1065	-0.02476	0.00504	-0.03464	-0.01488	-4.913	1.04E-06
1	rs12137445	215990733	A	ADDxstd_mixture_ab42	1065	-0.02418	0.005028	-0.03403	-0.01432	-4.809	1.74E-06
1	rs12141208	215992060	C	ADDxstd_mixture_ab42	1065	-0.02451	0.005206	-0.03472	-0.01431	-4.708	2.84E-06
1	rs12139361	215992211	A	ADDxstd_mixture_ab42	1065	-0.02418	0.005028	-0.03403	-0.01432	-4.809	1.74E-06
1	rs113042941	215994806	C	ADDxstd_mixture_ab42	1065	-0.02451	0.005206	-0.03472	-0.01431	-4.708	2.84E-06
1	rs4453013	215995439	A	ADDxstd_mixture_ab42	1065	-0.02418	0.005028	-0.03403	-0.01432	-4.809	1.74E-06
1	rs12138094	215996395	A	ADDxstd_mixture_ab42	1065	-0.02418	0.005028	-0.03403	-0.01432	-4.809	1.74E-06
1	rs79937377	215998400	A	ADDxstd_mixture_ab42	1065	-0.02418	0.005028	-0.03403	-0.01432	-4.809	1.74E-06
1	rs12141340	215999611	C	ADDxstd_mixture_ab42	1065	-0.02418	0.005028	-0.03403	-0.01432	-4.809	1.74E-06
1	rs11120650	215999733	T	ADDxstd_mixture_ab42	1065	-0.02418	0.005028	-0.03403	-0.01432	-4.809	1.74E-06
1	rs12117370	216002713	A	ADDxstd_mixture_ab42	1065	-0.02429	0.005065	-0.03422	-0.01437	-4.796	1.85E-06
1	rs79825305	216003716	A	ADDxstd_mixture_ab42	1065	-0.0242	0.005186	-0.03437	-0.01404	-4.666	3.46E-06
1	rs12409223	216004375	G	ADDxstd_mixture_ab42	1065	-0.02429	0.005065	-0.03422	-0.01437	-4.796	1.85E-06
1	rs112141149	216005846	G	ADDxstd_mixture_ab42	1065	-0.02429	0.005065	-0.03422	-0.01437	-4.796	1.85E-06
1	rs1934430	216008102	T	ADDxstd_mixture_ab42	1065	-0.02422	0.005066	-0.03415	-0.01429	-4.781	2.00E-06
1	rs1416842	216009305	A	ADDxstd_mixture_ab42	1065	-0.02459	0.005086	-0.03455	-0.01462	-4.834	1.54E-06
1	rs79789651	216010377	C	ADDxstd_mixture_ab42	1065	-0.02459	0.005086	-0.03455	-0.01462	-4.834	1.54E-06
1	rs41277194	216011408	C	ADDxstd_mixture_ab42	1065	-0.0245	0.005087	-0.03447	-0.01453	-4.816	1.68E-06
1	rs12402107	216012743	G	ADDxstd_mixture_ab42	1065	-0.0245	0.005087	-0.03447	-0.01453	-4.816	1.68E-06
1	rs76191410	216013040	A	ADDxstd_mixture_ab42	1065	-0.0245	0.005087	-0.03447	-0.01453	-4.816	1.68E-06
1	rs12123296	216013576	T	ADDxstd_mixture_ab42	1065	-0.02508	0.005099	-0.03507	-0.01508	-4.918	1.01E-06
1	rs12126534	216014657	A	ADDxstd_mixture_ab42	1065	-0.02508	0.005099	-0.03507	-0.01508	-4.918	1.01E-06
1	rs113393658	216015741	A	ADDxstd_mixture_ab42	1065	-0.02517	0.005098	-0.03517	-0.01518	-4.938	9.18E-07
1	rs146752822	216015809	A	ADDxstd_mixture_ab42	1065	-0.02517	0.005098	-0.03517	-0.01518	-4.938	9.18E-07
1	rs112735413	216016259	A	ADDxstd_mixture_ab42	1065	-0.02508	0.005099	-0.03507	-0.01508	-4.918	1.01E-06
1	rs75410548	216016299	C	ADDxstd_mixture_ab42	1065	-0.02508	0.005099	-0.03507	-0.01508	-4.918	1.01E-06
1	rs76992333	216016596	T	ADDxstd_mixture_ab42	1065	-0.02508	0.005099	-0.03507	-0.01508	-4.918	1.01E-06
1	rs12132798	216020536	G	ADDxstd_mixture_ab42	1065	-0.02517	0.005098	-0.03517	-0.01518	-4.938	9.18E-07
1	rs12126346	216021952	C	ADDxstd_mixture_ab42	1065	-0.0245	0.005087	-0.03447	-0.01453	-4.816	1.68E-06

1	rs12406554	216032737	T	ADDxstd_mixture_ab42	1065	-0.02512	0.005219	-0.03535	-0.01489	-4.814	1.70E-06
1	rs12129954	216033741	C	ADDxstd_mixture_ab42	1065	-0.02512	0.005219	-0.03535	-0.01489	-4.814	1.70E-06
3	rs4677869	122664907	T	ADDxstd_mixture_ab42	1065	0.01559	0.00283	0.01005	0.02114	5.51	4.50E-08
3	rs2288678	122667277	G	ADDxstd_mixture_ab42	1065	0.01602	0.002949	0.01024	0.0218	5.432	6.90E-08
3	rs4677982	122668294	C	ADDxstd_mixture_ab42	1065	0.01507	0.002929	0.009331	0.02081	5.146	3.17E-07
3	rs4677983	122669157	A	ADDxstd_mixture_ab42	1065	0.01494	0.002923	0.009213	0.02067	5.112	3.79E-07
3	rs4677984	122669629	T	ADDxstd_mixture_ab42	1065	0.01494	0.002923	0.009213	0.02067	5.112	3.79E-07
3	rs11706210	122669812	C	ADDxstd_mixture_ab42	1065	0.01546	0.002938	0.009697	0.02122	5.26	1.74E-07
3	rs4677870	122669854	C	ADDxstd_mixture_ab42	1065	0.01494	0.002923	0.009213	0.02067	5.112	3.79E-07
3	rs11707039	122670139	A	ADDxstd_mixture_ab42	1065	0.01546	0.002938	0.009697	0.02122	5.26	1.74E-07
3	rs35989119	122671081	C	ADDxstd_mixture_ab42	1065	0.01484	0.002925	0.009106	0.02057	5.073	4.62E-07
3	rs10934625	122671316	C	ADDxstd_mixture_ab42	1065	0.01483	0.002925	0.0091	0.02057	5.071	4.67E-07
3	rs62263260	122675327	T	ADDxstd_mixture_ab42	1065	0.01627	0.002849	0.01069	0.02185	5.711	1.46E-08
3	rs11707826	122676305	T	ADDxstd_mixture_ab42	1065	0.01624	0.002848	0.01066	0.02182	5.703	1.53E-08
3	rs10934626	122676523	T	ADDxstd_mixture_ab42	1065	0.01624	0.002848	0.01066	0.02182	5.703	1.53E-08
5	rs1363073	163418856	T	ADDxstd_mixture_ab42	1065	0.01073	0.002329	0.006162	0.01529	4.605	4.62E-06
6	rs116647170	150541084	T	ADDxstd_mixture_ab42	1065	0.04692	0.01049	0.02636	0.06749	4.472	8.57E-06
7	rs10279772	38706384	A	ADDxstd_mixture_ab42	1065	-0.009845	0.002193	-0.01414	-0.005547	-4.49	7.92E-06
7	rs10280089	38706817	A	ADDxstd_mixture_ab42	1065	-0.009845	0.002193	-0.01414	-0.005547	-4.49	7.92E-06
9	rs4130590	130107964	A	ADDxstd_mixture_ab42	1065	-0.008438	0.001861	-0.01209	-0.00479	-4.534	6.44E-06
9	rs7041940	130109746	T	ADDxstd_mixture_ab42	1065	-0.008438	0.001861	-0.01209	-0.00479	-4.534	6.44E-06
9	rs6478770	130112231	T	ADDxstd_mixture_ab42	1065	-0.008359	0.001862	-0.01201	-0.004709	-4.48	7.96E-06
11	rs938591	21075857	C	ADDxstd_mixture_ab42	1065	0.01013	0.002282	0.005657	0.0146	4.439	1.00E-05
11	rs10833448	21076108	T	ADDxstd_mixture_ab42	1065	0.0103	0.002282	0.00583	0.01477	4.515	7.05E-06
11	rs16917214	91976408	T	ADDxstd_mixture_ab42	1065	0.0101	0.002241	0.005708	0.01449	4.507	7.30E-06
11	rs16917218	91976564	A	ADDxstd_mixture_ab42	1065	0.0101	0.002241	0.005708	0.01449	4.507	7.30E-06
11	rs2054215	91976737	A	ADDxstd_mixture_ab42	1065	0.01012	0.002242	0.005724	0.01451	4.514	7.09E-06
11	rs12576325	91978130	T	ADDxstd_mixture_ab42	1065	0.01014	0.002244	0.005747	0.01454	4.521	6.85E-06
11	rs12786200	91979018	T	ADDxstd_mixture_ab42	1065	0.01014	0.002244	0.005747	0.01454	4.521	6.85E-06
11	rs12577025	91979120	A	ADDxstd_mixture_ab42	1065	0.0102	0.00224	0.005808	0.01459	4.553	5.90E-06
11	rs10501774	91979868	A	ADDxstd_mixture_ab42	1065	0.0102	0.00224	0.005808	0.01459	4.553	5.90E-06
11	rs1320636	91980524	A	ADDxstd_mixture_ab42	1065	0.0102	0.00224	0.005808	0.01459	4.553	5.90E-06
11	rs2212387	91981546	T	ADDxstd_mixture_ab42	1065	0.01014	0.002244	0.005747	0.01454	4.521	6.85E-06
11	rs7934732	91981618	T	ADDxstd_mixture_ab42	1065	0.01014	0.002244	0.005747	0.01454	4.521	6.85E-06
11	rs7935097	91981960	T	ADDxstd_mixture_ab42	1065	0.01025	0.002243	0.005856	0.01465	4.57	5.45E-06
11	rs1037731	91983763	G	ADDxstd_mixture_ab42	1065	0.01051	0.002216	0.006168	0.01486	4.743	2.39E-06
11	rs4753405	91984288	T	ADDxstd_mixture_ab42	1065	0.01025	0.002243	0.005856	0.01465	4.57	5.45E-06
11	rs4536170	91986020	C	ADDxstd_mixture_ab42	1065	0.01046	0.00222	0.006108	0.01481	4.711	2.79E-06

11	rs963960	91986620	T	ADDxstd_mixture_ab42	1065	0.01051	0.002216	0.006168	0.01486	4.743	2.39E-06
11	rs4542368	91988615	T	ADDxstd_mixture_ab42	1065	0.01052	0.002218	0.006169	0.01486	4.742	2.41E-06
11	rs12276382	91993712	C	ADDxstd_mixture_ab42	1065	0.01056	0.002235	0.006175	0.01494	4.723	2.64E-06
11	rs6483171	91996037	A	ADDxstd_mixture_ab42	1065	0.01044	0.002239	0.006053	0.01483	4.663	3.52E-06
11	rs7944251	91996431	A	ADDxstd_mixture_ab42	1065	0.01044	0.002239	0.006053	0.01483	4.663	3.52E-06
12	rs74592637	97545468	A	ADDxstd_mixture_ab42	1065	-0.02527	0.005628	-0.0363	-0.01424	-4.49	7.92E-06
12	rs2371519	97550185	G	ADDxstd_mixture_ab42	1065	-0.02527	0.005628	-0.0363	-0.01424	-4.49	7.92E-06
12	rs11108857	97553106	C	ADDxstd_mixture_ab42	1065	-0.02527	0.005628	-0.0363	-0.01424	-4.49	7.92E-06
16	rs7196791	25756588	T	ADDxstd_mixture_ab42	1065	-0.01332	0.002944	-0.01909	-0.00755	-4.524	6.75E-06
17	rs3110609	46753543	C	ADDxstd_mixture_ab42	1065	-0.009259	0.002049	-0.01327	-0.005243	-4.519	6.91E-06
17	rs3096644	46757575	T	ADDxstd_mixture_ab42	1065	-0.009195	0.002027	-0.01317	-0.005221	-4.535	6.41E-06

**Supplementary Table 11:** Summary of all coefficients from *APOE* interaction models			
Model: Baseline Hippoc. Vol. ~ Age at baseline MRI + sex + ***APOE*-ε4 x CSF Aβ-42**			
Predictor	B	SE	P value
Intercept	3.491	0.291	< 2.00e-16
Age at baseline MRI	-0.051	0.004	< 2.00e-16
Sex	-0.106	0.062	0.1
APOE-ε4	-0.008	0.086	0.923
CSF Aβ42	0.107	0.025	1.44e-5
APOE-ε4 x CSF Aβ42	0.111	0.031	0.0004
Model: Baseline Hippoc. Vol. ~ Age at baseline MRI + sex + ***APOE*-ε2 x CSF Aβ-42**			
Predictor	B	SE	P value
Intercept	3.345	0.283	< 2.00e-16
Age at baseline MRI	-0.050	0.004	< 2.00e-16
Sex	-0.093	0.062	0.134
APOE-ε2	0.006	0.109	0.955
CSF Aβ42	0.208	0.02	< 2.00e-16
APOE-ε2 x CSF Aβ42	-0.13	0.06	0.0435
Model: Annual Change Hippoc. Vol. ~ Age at baseline MRI + sex **+ *APOE*-ε4 x CSF Aβ-42**			
Predictor	B	SE	P value
Intercept	0.086	0.017	7.93e-7
Age at baseline MRI	-0.003	0.0002	< 2.00e-16
Sex	-0.0005	0.004	0.898
APOE-ε4	-0.034	0.005	0.494
CSF Aβ42	0.011	0.001	2.19e-13
APOE-ε4 x CSF Aβ42[b]	0.0056	0.002	0.0024
Model: Annual Change Hippoc. Vol. ~ Age at baseline MRI + sex + ***APOE*-ε2 x CSF Aβ-42**			
Predictor	B	SE	P value
Intercept	0.074	0.017	1.10e-5
Age at baseline MRI	-0.003	0.0002	< 2.00e-16
Sex	-0.0002	0.004	0.961
APOE-ε2	0.007	0.006	0.257
CSF Aβ42	0.016	0.001	< 2.00e-16
APOE-ε2x CSF Aβ42	-0.0054	0.004	0.152

**Supplementary Table 12:** All coefficients from rs62263260 x β-Amyloid model			
Model: Annual change of HV ~ Age at baseline MRI + sex + PC1-3 + rs62263260 x CSF Aβ-42			
Predictor	B	SE	P value
Intercept	0.0696	0.021	0.0013
Sex	-0.0007	0.005	0.877
Age at baseline MRI	-0.0026	0.0003	< 2.00e-16
PC1	-0.0523	0.114	0.646
PC2	-0.145	0.128	0.256
PC3	-0.0151	0.111	0.892
rs62263260	0.016	0.006	0.011
CSF Aβ42	0.014	0.002	< 2.00e-16
rs62263260 x CSF Aβ42	0.02621	0.0046	1.46e-08

**Supplementary Table 13.** Sensitivity Analyses for SNP x β-Amyloid GWAS								
Analysis	variant	chromosome	BP	allele	MAF	B	SE	P value
APOE4 + Scanner	rs62263260	3	122675327	T	0.121	0.01617	0.002761	6.30e-09
APOE4 + Scanner	rs11707826	3	122676305	T	0.122	0.01615	0.00276	6.54e-09
APOE4 + Scanner	rs10934626	3	122676523	T	0.122	0.01615	0.00276	6.54e-09
Mixed-Effects Regression	rs62263260	3	122675327	T	0.121	0.03302	0.00595	3.13e-08
Mixed-Effects Regression	rs11707826	3	122676305	T	0.122	0.03296	0.00595	3.27e-08
Mixed-Effects Regression	rs10934626	3	122676523	T	0.122	0.03294	0.00595	3.27e-08

Table S14. CSF AB Transcription Factor Binding Site Overlap

chr	rsID	pos	ref	alt	MAF	tag_rsID	tag_pos	tag_MAF	tag_name	R2	Dprime	motif_chr	motif_start	motif_end	tf_name	log_odds_score	strand	lative_snp_p	ref_pwm	alt_pwm	delta_pwm	ref_prob	alt_prob	motif_seq	p.fdr
chr3	rs2288678	122667277	A	G	0.081794	rs4677869	122664907	0.108179	SEMA5B-rs4677869	0.707308	0.981402	chr3	122667274	122667283	ETS1(ETS)	7	-	7	7.49974	2.639928	-4.85981	0.129	0.001	GCAGGATG	0.003333
chr3	rs2288678	122667277	A	G	0.081794	rs4677869	122664907	0.108179	SEMA5B-rs4677869	0.707308	0.981402	chr3	122667275	122667284	SPDEF(ETS)	8	+	3	7.607959	1.264079	-6.34388	0.569	0.001	ACATCCTG	0.003333
chr3	rs1093462	122671316	T	C	0.081794	rs4677869	122664907	0.108179	SEMA5B-rs4677869	0.707322	0.981411	chr3	122671310	122671321	Nur77(NR)	10	+	7	9.508289	2.603538	-6.90475	0.997	0.001	TAACCTTT	0.003333
chr3	rs1093462	122676523	C	T	0.085752	rs6226326	122675327	0.085752	SEMA5B-rs62263260	1	1	chr3	122676518	122676525	SCL(bHLH)	6	+	6	6.142697	1.036752	-5.10595	0.66	0.004	AGCAGCTT	0.01
chr3	rs3598911	122671081	A	C	0.081794	rs4677869	122664907	0.108179	SEMA5B-rs4677869	0.707322	0.981411	chr3	122671077	122671084	CRX(Homeo	5	+	5	4.855954	0.371412	-4.48454	0.975	0.011	ATTTATCC	0.022
chr3	rs2288678	122667277	A	G	0.081794	rs4677869	122664907	0.108179	SEMA5B-rs4677869	0.707308	0.981402	chr3	122667275	122667284	ETV1(ETS)	7	-	8	6.519016	4.889776	-1.62924	0.102	0.02	GGCAGGAT	0.033333
chr3	rs1093462	122676523	C	T	0.085752	rs6226326	122675327	0.085752	SEMA5B-rs62263260	1	1	chr3	122676517	122676524	SCL(bHLH)	6	-	2	6.477685	6.104669	-0.37302	0.379	0.261	AGCTGCTG	0.372857
chr3	rs1093462	122676523	C	T	0.085752	rs6226326	122675327	0.085752	SEMA5B-rs62263260	1	1	chr3	122676516	122676525	Tcf12(bHLH	7	+	8	7.484794	7.204834	-0.27996	0.43	0.325	ACAGCAGC	0.40625
chr3	rs4677870	122669854	C	G	0.081794	rs4677869	122664907	0.108179	SEMA5B-rs4677869	0.707322	0.981411	chr3	122669844	122669859	PAX5(Pairec	8	-	6	8.026829	8.852075	0.825246	0.262	0.598	GCAGTGAG	0.664444
chr3	rs4677870	122669854	C	G	0.081794	rs4677869	122664907	0.108179	SEMA5B-rs4677869	0.707322	0.981411	chr3	122669845	122669859	Pax8(Paired	10	+	10	9.654278	10.80432	1.150045	0.24	0.758	ATCATGGC	0.758

Table S15. MAGMA Gene-Test Results for SNP x AB42 GWAS for Hip. Slopes (subset to unadjusted p < 0.05)

GENE	CHR	START	STOP	NSNPS	NPARAM	N	ZSTAT	P	p.fdr
TOMM40	19	45394477	45406946	27	4	1065	4.4194	4.95E-06	0.086305
DPYD	1	97543299	98386615	1574	134	1065	4.1571	1.61E-05	0.140513
LOC101060400	17	46712166	46724875	37	7	1065	4.0241	2.86E-05	0.166253
USH2A	1	215796236	216596738	1692	165	1065	3.8066	7.04E-05	0.229768
MBTPS1	16	84087368	84150517	225	25	1065	3.7639	8.36E-05	0.229768
PACS1	11	65837747	66012218	225	14	1065	3.7639	8.36E-05	0.229768
HOXB9	17	46698518	46703835	14	4	1065	3.7394	9.22E-05	0.229768
ISM1	20	13202418	13281297	202	25	1065	3.6414	1.36E-04	0.295603
COBL	7	51083909	51384515	480	40	1065	3.5566	1.88E-04	0.363955
CDK5RAP3	17	46047894	46059152	28	4	1065	3.5124	2.22E-04	0.387225
NRL	14	24547902	24584223	27	7	1065	3.4523	2.78E-04	0.405547
HOXB3	17	46626232	46667634	79	15	1065	3.4512	2.79E-04	0.405547
IL12RB2	1	67773047	67862583	199	15	1065	3.4036	3.32E-04	0.425523
APOE	19	45409039	45412650	4	2	1065	3.3963	3.42E-04	0.425523
CAPN10	2	241526133	241538526	49	5	1065	3.2482	5.81E-04	0.636258
SP100	2	231280871	231410317	338	36	1065	3.2306	6.18E-04	0.636258
ZNF695	1	247108849	247171395	171	22	1065	3.2294	6.20E-04	0.636258
ZNF670	1	247197940	247242115	185	16	1065	3.1827	7.30E-04	0.703469
LTBR	12	6484534	6500737	6	2	1065	3.1684	7.66E-04	0.703469
PPA1	10	71962586	71993667	97	13	1065	3.153	8.08E-04	0.704738
PRR15L	17	46029333	46035252	18	5	1065	3.1321	8.68E-04	0.715969
HOXB13	17	46802125	46806111	7	4	1065	3.1204	9.03E-04	0.715969
OR7A17	19	14991238	14992167	6	3	1065	3.0935	9.89E-04	0.729106
SERBP1	1	67873493	67896123	14	3	1065	3.0892	1.00E-03	0.729106
DCAF11	14	24583906	24594451	14	3	1065	3.032	1.21E-03	0.75895
ZNF302	19	35168553	35177302	28	5	1065	3.0303	1.22E-03	0.75895

GRTP1	13	113978505	114018463	78	10	1065	3.0267	1.24E-03	0.75895
MICU3	8	16884747	16980148	234	24	1065	3.0164	1.28E-03	0.75895
CCR3	3	46283872	46308197	54	7	1065	2.9855	1.42E-03	0.75895
SPATS2L	2	201170604	201346986	346	45	1065	2.9518	1.58E-03	0.75895
KDM4D	11	94706845	94732678	48	9	1065	2.9355	1.67E-03	0.75895
LSM5	7	32524945	32534870	39	6	1065	2.9331	1.68E-03	0.75895
GAL3ST3	11	65808236	65816651	13	2	1065	2.908	1.82E-03	0.75895
APC	5	112043202	112181936	224	20	1065	2.9076	1.82E-03	0.75895
NTNG2	9	135037334	135118224	94	13	1065	2.9059	1.83E-03	0.75895
TLDC1	16	84509966	84538366	151	23	1065	2.9041	1.84E-03	0.75895
PIK3CD	1	9711789	9789172	48	9	1065	2.8853	1.96E-03	0.75895
CNIH2	11	66045672	66051685	4	2	1065	2.8844	1.96E-03	0.75895
YIF1A	11	66052051	66056638	5	2	1065	2.8827	1.97E-03	0.75895
SSC4D	7	76018646	76039012	70	7	1065	2.8816	1.98E-03	0.75895
ANKMY1	2	241418839	241500472	214	33	1065	2.871	2.05E-03	0.75895
CDC34	19	531720	542087	8	1	1065	2.8483	2.20E-03	0.75895
MAATS1	3	119421869	119485949	94	11	1065	2.8437	2.23E-03	0.75895
GML	8	143916217	143928262	44	6	1065	2.8416	2.24E-03	0.75895
OR10H2	19	15838834	15839862	5	2	1065	2.8415	2.25E-03	0.75895
PLA1A	3	119316695	119348658	101	8	1065	2.8408	2.25E-03	0.75895
RAB27A	15	55495164	55582034	251	27	1065	2.8374	2.27E-03	0.75895
SCN8A	12	51984050	52206648	282	21	1065	2.8371	2.28E-03	0.75895
PDGFC	4	157682763	157892546	212	17	1065	2.8314	2.32E-03	0.75895
ZNF79	9	130186653	130209525	47	5	1065	2.8292	2.33E-03	0.75895
FIZ1	19	56102737	56110893	10	3	1065	2.8211	2.39E-03	0.75895
HERC6	4	89299838	89364249	24	5	1065	2.808	2.49E-03	0.75895
ZNF716	7	57509883	57533265	85	4	1065	2.8066	2.50E-03	0.75895
SAMD4A	14	55033815	55260033	372	83	1065	2.8057	2.51E-03	0.75895
CCDC3	10	12938625	13141762	729	96	1065	2.7976	2.57E-03	0.75895
UGT3A1	5	35953191	36066639	276	33	1065	2.7913	2.62E-03	0.75895
IL1R2	2	102608306	102644885	115	8	1065	2.7684	2.82E-03	0.75895
RNF125	18	29598445	29653154	70	8	1065	2.7608	2.88E-03	0.75895
HOXB6	17	46673099	46682343	16	6	1065	2.75	2.98E-03	0.75895

PNLIP	10	118305428	118327367	25	3	1065	2.7487	2.99E-03	0.75895
SCN1B	19	35521555	35531353	7	1	1065	2.7413	3.06E-03	0.75895
NAPB	20	23355156	23402156	157	13	1065	2.7387	3.08E-03	0.75895
DVL2	17	7128661	7137867	9	3	1065	2.7363	3.11E-03	0.75895
PRAC1	17	46799081	46799882	1	1	1065	2.732	3.15E-03	0.75895
SPPL3	12	121200313	121342155	381	31	1065	2.7279	3.19E-03	0.75895
MRPL18	6	160211492	160219461	35	4	1065	2.7202	3.26E-03	0.75895
INSR	19	7112266	7294011	447	55	1065	2.7179	3.29E-03	0.75895
LMAN1	18	56995055	57026508	77	8	1065	2.7178	3.29E-03	0.75895
SRSF11	1	70671365	70717703	39	4	1065	2.7174	3.29E-03	0.75895
MTNR1B	11	92702789	92715948	25	4	1065	2.7117	3.35E-03	0.75895
TAAR9	6	132859427	132860475	4	2	1064	2.7083	3.38E-03	0.75895
RPL12	9	130209953	130213711	7	1	1065	2.7077	3.39E-03	0.75895
GJB2	13	20761602	20768605	14	3	1065	2.7068	3.40E-03	0.75895
TAS2R42	12	11338599	11339543	8	2	1065	2.7045	3.42E-03	0.75895
SCRIB	8	144873090	144897890	30	4	1065	2.7028	3.44E-03	0.75895
CMC1	3	28282838	28361264	115	11	1065	2.6979	3.49E-03	0.75895
SAXO1	9	18927891	19033256	498	33	1065	2.6892	3.58E-03	0.75895
ESM1	5	54273695	54281414	13	2	1065	2.6851	3.63E-03	0.75895
LMTK3	19	48988528	49016446	26	2	1065	2.684	3.64E-03	0.75895
ZP3	7	76026841	76071388	142	17	1065	2.681	3.67E-03	0.75895
CPLX4	18	56962634	56985881	60	11	1065	2.6805	3.68E-03	0.75895
CARD6	5	40841410	40855456	20	5	1065	2.6774	3.71E-03	0.75895
ZCWPW2	3	28390372	28566633	188	12	1065	2.677	3.71E-03	0.75895
DNAH6	2	84743579	85046713	556	21	1065	2.6713	3.78E-03	0.75895
FAM32A	19	16296235	16302857	13	3	1065	2.6559	3.96E-03	0.75895
APOBEC3A	22	39348755	39359189	6	2	1065	2.6535	3.98E-03	0.75895
KRT78	12	53231588	53242778	18	4	1065	2.6494	4.03E-03	0.75895
SEMA5B	3	122628040	122747452	309	43	1065	2.6486	4.04E-03	0.75895
PPP6R3	11	68228186	68382802	178	7	1065	2.6486	4.04E-03	0.75895
CDKN1C	11	2904448	2907063	1	1	1065	2.6485	4.04E-03	0.75895
MLEC	12	121124949	121139667	35	8	1065	2.6385	4.16E-03	0.75895
C15orf62	15	41062159	41064647	2	1	1064	2.6377	4.17E-03	0.75895

SEL1L	14	81937891	82000205	41	7	1065	2.6327	4.24E-03	0.75895
C17orf96	17	36827956	36831187	3	2	1065	2.6317	4.25E-03	0.75895
LRSAM1	9	130213765	130265780	104	12	1065	2.6292	4.28E-03	0.75895
SLC2A8	9	130159417	130170177	21	4	1065	2.6278	4.30E-03	0.75895
IL22RA2	6	137464957	137494785	74	12	1065	2.6263	4.32E-03	0.75895
C15orf53	15	38988799	38992239	19	4	1065	2.612	4.50E-03	0.75895
C1orf27	1	186344890	186390503	65	10	1065	2.6116	4.51E-03	0.75895
KLC2	11	66024765	66035332	12	3	1065	2.6049	4.59E-03	0.75895
PUF60	8	144898514	144911556	15	2	1065	2.5962	4.71E-03	0.75895
AFF3	2	100162326	100759037	936	73	1065	2.5954	4.72E-03	0.75895
AGA	4	178351928	178363657	25	7	1065	2.5818	4.91E-03	0.75895
FITM1	14	24600675	24602058	2	1	1065	2.5817	4.92E-03	0.75895
WDR11	10	122610687	122669038	185	9	1065	2.5811	4.92E-03	0.75895
LONRF1	8	12579406	12612992	72	6	1065	2.5755	5.00E-03	0.75895
AHCTF1	1	247002400	247094726	127	13	1065	2.5738	5.03E-03	0.75895
HERC5	4	89378181	89427334	15	3	1065	2.5729	5.04E-03	0.75895
CWC15	11	94695787	94706776	23	6	1065	2.5723	5.05E-03	0.75895
TMEM159	16	21169703	21191937	35	6	1065	2.5695	5.09E-03	0.75895
ZPR1	11	116649276	116658739	20	6	1065	2.5686	5.11E-03	0.75895
RSL24D1	15	55473512	55489231	48	6	1065	2.5672	5.13E-03	0.75895
C16orf87	16	46835959	46865074	13	1	1065	2.566	5.14E-03	0.75895
DNER	2	230222345	230579286	909	96	1065	2.5613	5.21E-03	0.75895
COLCA2	11	111169271	111179460	23	4	1065	2.5604	5.23E-03	0.75895
BTN1A1	6	26500577	26510653	18	3	1065	2.5543	5.32E-03	0.75895
CHCHD5	2	113342023	113346617	10	3	1065	2.5487	5.41E-03	0.75895
EMC9	14	24608081	24610837	2	1	1065	2.5468	5.44E-03	0.75895
TOR1AIP1	1	179851177	179889212	87	9	1065	2.5446	5.47E-03	0.75895
C4BPB	1	207262210	207273338	14	4	1065	2.5378	5.58E-03	0.75895
KRT8	12	53290971	53343650	143	18	1065	2.5305	5.69E-03	0.75895
DNAAF1	16	84178865	84211524	116	18	1065	2.5232	5.81E-03	0.75895
ACD	16	67691415	67694718	4	2	1065	2.5184	5.90E-03	0.75895
RASGEF1B	4	82347547	82393082	63	9	1065	2.5162	5.93E-03	0.75895
SIPA1L1	14	71788108	72207761	424	25	1065	2.5129	5.99E-03	0.75895

MAST1	19	12949259	12985766	49	3	1065	2.5091	6.05E-03	0.75895
TPT1	13	45911005	45915505	9	2	1065	2.5064	6.10E-03	0.75895
HMGN4	6	26538572	26547165	15	2	1065	2.5057	6.11E-03	0.75895
KIF5B	10	32297938	32345371	74	7	1065	2.5044	6.13E-03	0.75895
CCDC141	2	179694484	179914841	519	59	1065	2.5021	6.17E-03	0.75895
CPT1B	22	51007290	51017096	38	7	1065	2.5011	6.19E-03	0.75895
ZFP57	6	29640169	29644931	23	8	1065	2.5007	6.20E-03	0.75895
ACTA1	1	229566992	229569858	3	1	1065	2.4997	6.21E-03	0.75895
COX19	7	1004486	1015235	14	3	1065	2.499	6.23E-03	0.75895
MTERF2	12	107371069	107380944	19	7	1065	2.4966	6.27E-03	0.75895
C14orf28	14	45366433	45376460	16	2	1064	2.4955	6.29E-03	0.75895
DCLK3	3	36753913	36781352	45	10	1065	2.4954	6.29E-03	0.75895
ALAD	9	116148592	116163618	9	2	1065	2.4922	6.35E-03	0.75895
CA1	8	86240458	86290342	62	10	1065	2.4901	6.38E-03	0.75895
CEP85L	6	118781935	119031238	584	32	1065	2.4901	6.39E-03	0.75895
CADM2	3	85008133	86123579	2425	88	1065	2.4896	6.39E-03	0.75895
ZNF850	19	37234380	37263717	63	8	1065	2.4893	6.40E-03	0.75895
AP2M1	3	183892634	183901879	16	5	1065	2.4862	6.46E-03	0.75895
DTX3L	3	122283085	122294050	12	5	1065	2.4861	6.46E-03	0.75895
DZIP3	3	108308337	108413693	118	11	1065	2.4757	6.65E-03	0.75895
AOAH	7	36552549	36764154	489	61	1065	2.4719	6.72E-03	0.75895
TMEM55B	14	20926012	20929637	1	1	1065	2.4691	6.77E-03	0.75895
C2CD3	11	73723759	73882064	203	14	1065	2.4636	6.88E-03	0.75895
SPNS2	17	4402129	4443228	54	7	1065	2.4607	6.93E-03	0.75895
CHKB	22	51017387	51021428	12	4	1065	2.4587	6.97E-03	0.75895
C4orf22	4	81256861	81884910	916	45	1065	2.4586	6.97E-03	0.75895
TMEM26	10	63166401	63213208	103	15	1065	2.4581	6.98E-03	0.75895
TAAR8	6	132873832	132874860	1	1	1065	2.455	7.05E-03	0.75895
AQP12B	2	241615835	241622317	3	2	1065	2.4547	7.05E-03	0.75895
TAS2R50	12	11138512	11139511	3	2	1065	2.4532	7.08E-03	0.75895
PRR4	12	10998448	11002075	8	3	1065	2.4483	7.18E-03	0.75895
HOXB5	17	46668619	46671103	8	4	1065	2.4475	7.19E-03	0.75895
KLK12	19	51532348	51538486	6	1	1065	2.4402	7.34E-03	0.75895

MYL4	17	45266728	45301045	60	12	1065	2.4352	7.44E-03	0.75895
RAVER2	1	65210778	65298915	133	13	1065	2.4324	7.50E-03	0.75895
CREB1	2	208394616	208470284	75	6	1065	2.4304	7.54E-03	0.75895
POPDC2	3	119360899	119379437	64	8	1065	2.4301	7.55E-03	0.75895
EDIL3	5	83236414	83680685	660	74	1065	2.4284	7.58E-03	0.75895
OR2B11	1	247614331	247615284	4	1	1065	2.4281	7.59E-03	0.75895
GZF1	20	23342769	23353683	28	6	1065	2.4235	7.69E-03	0.75895
TM9SF2	13	100153628	100216302	114	15	1065	2.423	7.70E-03	0.75895
DNAJC17	15	41060067	41099676	48	5	1065	2.4201	7.76E-03	0.75895
ACAP1	17	7239848	7254797	17	5	1065	2.4173	7.82E-03	0.75895
LTBP4	19	41099072	41135725	83	9	1065	2.4171	7.82E-03	0.75895
COLCA1	11	111164114	111177104	30	4	1065	2.4146	7.88E-03	0.75895
HOXB7	17	46684594	46688383	11	4	1065	2.4141	7.89E-03	0.75895
TPM4	19	16178317	16213815	32	4	1065	2.4134	7.90E-03	0.75895
MXRA7	17	74669748	74707056	106	11	1065	2.4099	7.98E-03	0.75895
SCG2	2	224461658	224467217	3	1	1065	2.4084	8.01E-03	0.75895
FBXO28	1	224301789	224349749	89	5	1065	2.4082	8.02E-03	0.75895
NLRP5	19	56511092	56573176	259	43	1065	2.4074	8.03E-03	0.75895
IL31RA	5	55147207	55218682	156	21	1065	2.4072	8.04E-03	0.75895
SNAP23	15	42783442	42825259	46	4	1065	2.4067	8.05E-03	0.75895
TAS2R7	12	10954131	10955226	1	1	1065	2.4051	8.08E-03	0.75895
NMRAL1	16	4511690	4524896	39	3	1065	2.4007	8.18E-03	0.75895
PRRG2	19	50084574	50094272	21	3	1065	2.398	8.24E-03	0.75895
PFN2	3	149682691	149688741	7	3	1065	2.3972	8.26E-03	0.75895
PNLDC1	6	160221281	160241736	47	8	1065	2.3962	8.28E-03	0.75895
TPR	1	186280784	186344883	115	11	1065	2.3959	8.29E-03	0.75895
IGL	22	22380474	23265085	738	73	1065	2.3953	8.30E-03	0.75895
POLR3D	8	22102619	22108680	16	5	1065	2.3931	8.35E-03	0.75895
TAF1C	16	84211453	84220676	44	9	1065	2.392	8.38E-03	0.75895
VILL	3	38032217	38048676	41	5	1065	2.3912	8.40E-03	0.75895
C8orf22	8	49966895	49988642	29	4	1065	2.3871	8.49E-03	0.75895
ONECUT2	18	55102917	55158530	137	13	1065	2.3834	8.58E-03	0.75895
ARHGEF5	7	144052489	144077725	8	1	1065	2.3825	8.60E-03	0.75895

DRG2	17	17991181	18011299	30	4	1065	2.3791	8.68E-03	0.75895
SLC3A2	11	62623484	62656355	17	4	1065	2.3779	8.71E-03	0.75895
CCR5	3	46411633	46417697	13	3	1065	2.3752	8.77E-03	0.75895
CCRL2	3	46448721	46454488	15	3	1065	2.3734	8.81E-03	0.75895
IRG1	13	77522694	77532777	19	6	1065	2.3719	8.85E-03	0.75895
PLD5	1	242251689	242687998	1238	120	1065	2.3678	8.95E-03	0.75895
SLC30A8	8	117962512	118188953	333	26	1065	2.3664	8.98E-03	0.75895
PHF23	17	7138347	7142825	3	1	1065	2.3653	9.01E-03	0.75895
ATP6V1C2	2	10861775	10925236	161	22	1065	2.3646	9.03E-03	0.75895
ZNF792	19	35447078	35455139	15	3	1065	2.3644	9.03E-03	0.75895
OR4F15	15	102358390	102359328	1	1	1065	2.3639	9.04E-03	0.75895
GNA15	19	3136191	3163766	55	9	1065	2.3627	9.07E-03	0.75895
PRF1	10	72357104	72362531	10	3	1065	2.3617	9.10E-03	0.75895
SNRPB	20	2442281	2451499	10	2	1065	2.3616	9.10E-03	0.75895
DNASE2	19	12986025	12992409	5	1	1065	2.3602	9.13E-03	0.75895
FGFR1OP	6	167412805	167458064	224	9	1065	2.3597	9.14E-03	0.75895

Table S16. MAGMA Gene-Test Results for SNP x AB42 GWAS for Hip. Slopes, covaried for APOE-e4 (subset to unadjusted $p < 0.05$)

GENE	CHR	START	STOP	NSNPS	NPARAM	N	ZSTAT	P	p.fdr
DPYD	1	97543299	98386615	1574	134	1058	4.5405	2.81E-06	0.048946
COBL	7	51083909	51384515	480	40	1058	3.7376	9.29E-05	0.373287
ISM1	20	13202418	13281297	202	25	1058	3.7235	9.82E-05	0.373287
PACS1	11	65837747	66012218	225	14	1058	3.7185	1.00E-04	0.373287
LOC10106C	17	46712166	46724875	37	7	1058	3.6495	1.31E-04	0.373287
TLDC1	16	84509966	84538366	151	23	1058	3.6388	1.37E-04	0.373287
MICU3	8	16884747	16980148	234	24	1058	3.6156	1.50E-04	0.373287
USH2A	1	215796236	216596738	1692	165	1058	3.5375	2.02E-04	0.440363
HOXB9	17	46698518	46703835	14	4	1058	3.465	2.65E-04	0.471922
OR10H2	19	15838834	15839862	5	2	1058	3.4411	2.90E-04	0.471922
IL12RB2	1	67773047	67862583	199	15	1058	3.4338	2.98E-04	0.471922
HOXB3	17	46626232	46667634	79	15	1058	3.3631	3.85E-04	0.539413
TOMM40	19	45394477	45406946	27	4	1058	3.3399	4.19E-04	0.539413
RAB27A	15	55495164	55582034	251	27	1058	3.3308	4.33E-04	0.539413
CAPN10	2	241526133	241538526	49	5	1058	3.269	5.40E-04	0.573415
ZNF670	1	247197940	247242115	185	16	1058	3.2447	5.88E-04	0.573415
NRL	14	24547902	24584223	27	7	1058	3.2054	6.74E-04	0.573415
CDK5RAP3	17	46047894	46059152	28	4	1058	3.1859	7.22E-04	0.573415
ZNF695	1	247108849	247171395	171	22	1058	3.1772	7.43E-04	0.573415
SERBP1	1	67873493	67896123	14	3	1058	3.1713	7.59E-04	0.573415
ACD	16	67691415	67694718	4	2	1058	3.1601	7.89E-04	0.573415
IGL	22	22380474	23265085	738	73	1058	3.1539	8.06E-04	0.573415
KDM4D	11	94706845	94732678	48	9	1058	3.1492	8.19E-04	0.573415
AFF3	2	100162326	100759037	936	73	1058	3.1487	8.20E-04	0.573415
VILL	3	38032217	38048676	41	5	1058	3.131	8.71E-04	0.573415
ENDOV	17	78388965	78411886	37	7	1058	3.1283	8.79E-04	0.573415
DCAF11	14	24583906	24594451	14	3	1058	3.1162	9.16E-04	0.573415
FIZ1	19	56102737	56110893	10	3	1058	3.1107	9.33E-04	0.573415

LTBR	12	6484534	6500737	6	2	1058	3.1002	9.67E-04	0.573415
SPNS2	17	4402129	4443228	54	7	1058	3.0927	9.92E-04	0.573415
IL1R2	2	102608306	102644885	115	8	1058	3.0846	1.02E-03	0.573415
CCR3	3	46283872	46308197	54	7	1058	3.0398	1.18E-03	0.602321
PPA1	10	71962586	71993667	97	13	1058	3.0389	1.19E-03	0.602321
ZNF302	19	35168553	35177302	28	5	1058	3.0362	1.20E-03	0.602321
SP100	2	231280871	231410317	338	36	1058	3.0059	1.32E-03	0.602321
CMC1	3	28282838	28361264	115	11	1058	3.0049	1.33E-03	0.602321
APC	5	112043202	112181936	224	20	1058	2.9975	1.36E-03	0.602321
SCRIB	8	144873090	144897890	30	4	1058	2.9963	1.37E-03	0.602321
SAMD4A	14	55033815	55260033	372	83	1058	2.987	1.41E-03	0.602321
NAPB	20	23355156	23402156	157	13	1058	2.9691	1.49E-03	0.602321
GML	8	143916217	143928262	44	6	1058	2.9687	1.50E-03	0.602321
RSL24D1	15	55473512	55489231	48	6	1058	2.965	1.51E-03	0.602321
GAL3ST3	11	65808236	65816651	13	2	1058	2.9548	1.56E-03	0.602321
C17orf96	17	36827956	36831187	3	2	1058	2.9548	1.56E-03	0.602321
TM9SF2	13	100153628	100216302	114	15	1058	2.9515	1.58E-03	0.602321
PIK3CD	1	9711789	9789172	48	9	1058	2.9501	1.59E-03	0.602321
C15orf53	15	38988799	38992239	19	4	1058	2.9417	1.63E-03	0.605574
C14orf28	14	45366433	45376460	16	2	1057	2.934	1.67E-03	0.607928
LMAN1	18	56995055	57026508	77	8	1058	2.9085	1.82E-03	0.646349
AGA	4	178351928	178363657	25	7	1058	2.8924	1.91E-03	0.65373
EDIL3	5	83236414	83680685	660	74	1058	2.8924	1.91E-03	0.65373
PLA1A	3	119316695	119348658	101	8	1058	2.8789	2.00E-03	0.659488
SPATS2L	2	201170604	201346986	346	45	1058	2.8698	2.05E-03	0.659488
PUF60	8	144898514	144911556	15	2	1058	2.8684	2.06E-03	0.659488
NRXN1	2	50145643	51259674	2995	193	1058	2.8613	2.11E-03	0.659488
DNAH8	6	38683117	38998574	1015	58	1058	2.8601	2.12E-03	0.659488
WDFY1	2	224740065	224810104	143	6	1058	2.8415	2.25E-03	0.678027
TAAR9	6	132859427	132860475	4	2	1057	2.8383	2.27E-03	0.678027
MAST1	19	12949259	12985766	49	3	1058	2.8212	2.39E-03	0.678027
CNBD1	8	87878676	88394955	1148	71	1058	2.8196	2.40E-03	0.678027
FAM32A	19	16296235	16302857	13	3	1058	2.8043	2.52E-03	0.678027
SCG2	2	224461658	224467217	3	1	1058	2.8037	2.53E-03	0.678027
HERC6	4	89299838	89364249	24	5	1058	2.7971	2.58E-03	0.678027

POP5	12	121016848	121019201	9	3	1058	2.7953	2.59E-03	0.678027
AOAH	7	36552549	36764154	489	61	1058	2.7863	2.67E-03	0.678027
CPLX4	18	56962634	56985881	60	11	1058	2.7832	2.69E-03	0.678027
UGT3A1	5	35953191	36066639	276	33	1058	2.7796	2.72E-03	0.678027
DHRS12	13	52330614	52379667	62	13	1058	2.7783	2.73E-03	0.678027
TAS2R42	12	11338599	11339543	8	2	1058	2.7715	2.79E-03	0.678027
SAXO1	9	18927891	19033256	498	33	1058	2.7672	2.83E-03	0.678027
APOE	19	45409039	45412650	4	2	1058	2.766	2.84E-03	0.678027
CWC15	11	94695787	94706776	23	6	1058	2.762	2.87E-03	0.678027
MLEC	12	121124949	121139667	35	8	1058	2.7487	2.99E-03	0.678027
LONRF1	8	12579406	12612992	72	6	1058	2.7409	3.06E-03	0.678027
TOR1AIP1	1	179851177	179889212	87	9	1058	2.7323	3.14E-03	0.678027
OR7A17	19	14991238	14992167	6	3	1058	2.721	3.25E-03	0.678027
AQP12B	2	241615835	241622317	3	2	1058	2.721	3.25E-03	0.678027
SCN1B	19	35521555	35531353	7	1	1058	2.7209	3.26E-03	0.678027
ZCWPW2	3	28390372	28566633	188	12	1058	2.7176	3.29E-03	0.678027
CARD6	5	40841410	40855456	20	5	1058	2.7163	3.30E-03	0.678027
ZPR1	11	116649276	116658739	20	6	1058	2.714	3.32E-03	0.678027
HOXB13	17	46802125	46806111	7	4	1058	2.7127	3.34E-03	0.678027
NTNG2	9	135037334	135118224	94	13	1058	2.7098	3.37E-03	0.678027
LSM5	7	32524945	32534870	39	6	1058	2.7096	3.37E-03	0.678027
CNIH2	11	66045672	66051685	4	2	1058	2.707	3.39E-03	0.678027
PLA2G4C	19	48551100	48614109	208	31	1058	2.7028	3.44E-03	0.678027
COX19	7	1004486	1015235	14	3	1058	2.698	3.49E-03	0.678027
RIPK3	14	24805227	24809242	16	2	1058	2.6969	3.50E-03	0.678027
SRSF11	1	70671365	70717703	39	4	1058	2.6923	3.55E-03	0.678027
FBXO28	1	224301789	224349749	89	5	1058	2.6915	3.56E-03	0.678027
CCR5	3	46411633	46417697	13	3	1058	2.6896	3.58E-03	0.678027
MAATS1	3	119421869	119485949	94	11	1058	2.6853	3.62E-03	0.678027
GATC	12	120884241	120901556	40	8	1058	2.6794	3.69E-03	0.678027
SLC3A2	11	62623484	62656355	17	4	1058	2.6724	3.77E-03	0.678027
C4orf22	4	81256861	81884910	916	45	1058	2.6657	3.84E-03	0.678027
DZIP3	3	108308337	108413693	118	11	1058	2.6637	3.86E-03	0.678027
C7orf50	7	1036623	1177911	509	35	1058	2.6621	3.88E-03	0.678027
PLD5	1	242251689	242687998	1238	120	1058	2.662	3.88E-03	0.678027

SPPL3	12	121200313	121342155	381	31	1058	2.6599	3.91E-03	0.678027
GZF1	20	23342769	23353683	28	6	1058	2.6482	4.05E-03	0.678027
MKRN2OS	3	12581280	12586963	11	3	1058	2.6442	4.09E-03	0.678027
CPT1B	22	51007290	51017096	38	7	1058	2.642	4.12E-03	0.678027
MBTPS1	16	84087368	84150517	225	25	1058	2.6386	4.16E-03	0.678027
PRR15L	17	46029333	46035252	18	5	1058	2.6281	4.29E-03	0.678027
YIF1A	11	66052051	66056638	5	2	1058	2.6253	4.33E-03	0.678027
RLTPR	16	67679030	67691472	14	3	1058	2.6221	4.37E-03	0.678027
FAM160B1	10	116581503	116659586	197	14	1058	2.6184	4.42E-03	0.678027
C4BPB	1	207262210	207273338	14	4	1058	2.6076	4.56E-03	0.678027
IL22RA2	6	137464957	137494785	74	12	1058	2.6027	4.62E-03	0.678027
KCND3	1	112318444	112532147	564	90	1058	2.597	4.70E-03	0.678027
HACD1	10	17631031	17659383	92	14	1058	2.5966	4.71E-03	0.678027
DVL2	17	7128661	7137867	9	3	1058	2.5944	4.74E-03	0.678027
SEMA5B	3	122628040	122747452	309	43	1058	2.5932	4.75E-03	0.678027
GJB2	13	20761602	20768605	14	3	1058	2.5915	4.78E-03	0.678027
OR10H3	19	15852203	15853153	7	3	1058	2.5901	4.80E-03	0.678027
ENKD1	16	67696850	67700628	1	1	1058	2.5889	4.81E-03	0.678027
ACAP1	17	7239848	7254797	17	5	1058	2.5885	4.82E-03	0.678027
CDC34	19	531720	542087	8	1	1058	2.5877	4.83E-03	0.678027
BTN1A1	6	26500577	26510653	18	3	1058	2.5876	4.83E-03	0.678027
C7	5	40909599	40983041	265	26	1058	2.5875	4.83E-03	0.678027
SEMA6B	19	4542600	4559771	14	5	1058	2.5791	4.95E-03	0.678027
DTX3L	3	122283085	122294050	12	5	1058	2.5717	5.06E-03	0.678027
NPAP1	15	24920541	24928593	20	5	1058	2.5714	5.06E-03	0.678027
HERC5	4	89378181	89427334	15	3	1058	2.5709	5.07E-03	0.678027
SCN8A	12	51984050	52206648	282	21	1058	2.5632	5.19E-03	0.678027
NXF1	11	62559597	62572964	6	3	1058	2.5609	5.22E-03	0.678027
LTBP4	19	41099072	41135725	83	9	1058	2.5556	5.30E-03	0.678027
ALAD	9	116148592	116163618	9	2	1058	2.5525	5.35E-03	0.678027
ARHGEF5	7	144052489	144077725	8	1	1058	2.5506	5.38E-03	0.678027
DNASE2	19	12986025	12992409	5	1	1058	2.5497	5.39E-03	0.678027
PHOX2A	11	71950121	71955220	4	1	1058	2.5484	5.41E-03	0.678027
CHKB	22	51017387	51021428	12	4	1058	2.5391	5.56E-03	0.678027
OR10H1	19	15917817	15918936	3	2	1058	2.5375	5.58E-03	0.678027

DNAH6	2	84743579	85046713	556	21	1058	2.5354	5.62E-03	0.678027
PPP4R1	18	9546789	9614605	157	17	1058	2.5343	5.63E-03	0.678027
OR2B11	1	247614331	247615284	4	1	1058	2.5342	5.63E-03	0.678027
EPSTI1	13	43460524	43566407	282	39	1058	2.5334	5.65E-03	0.678027
HMGN4	6	26538572	26547165	15	2	1058	2.5325	5.66E-03	0.678027
SVIL	10	29746274	30025864	968	104	1058	2.5311	5.68E-03	0.678027
LDLR	19	11200037	11244506	127	17	1058	2.53	5.70E-03	0.678027
PAQR6	1	156213112	156217908	3	2	1058	2.5289	5.72E-03	0.678027
OSBP2	22	31089769	31303811	434	49	1058	2.524	5.80E-03	0.678027
IRS1	2	227596033	227664545	109	7	1058	2.5225	5.83E-03	0.678027
SGSM1	22	25202136	25322813	349	50	1058	2.522	5.83E-03	0.678027
KLF17	1	44513987	44600812	257	27	1058	2.5207	5.86E-03	0.678027
TMPRSS2	21	42836236	42880085	139	18	1058	2.5204	5.86E-03	0.678027
RBP1	3	139236276	139258671	54	11	1058	2.519	5.88E-03	0.678027
MRPL16	11	59573608	59578345	2	1	1058	2.5173	5.91E-03	0.678027
EMC9	14	24608081	24610837	2	1	1058	2.5152	5.95E-03	0.678027
CTCF	16	67596310	67673088	63	7	1058	2.5149	5.95E-03	0.678027
KRT78	12	53231588	53242778	18	4	1058	2.5138	5.97E-03	0.678027
SYNPO2L	10	75404639	75415832	11	2	1058	2.5133	5.98E-03	0.678027
NAV3	12	78224685	78606790	1011	89	1058	2.5123	6.00E-03	0.678027
MTERF2	12	107371069	107380944	19	7	1058	2.5112	6.02E-03	0.678027
HOXB6	17	46673099	46682343	16	6	1058	2.5105	6.03E-03	0.678027
MXRA7	17	74669748	74707056	106	11	1058	2.5074	6.08E-03	0.678027
C1orf27	1	186344890	186390503	65	10	1058	2.5048	6.13E-03	0.678027
KLC2	11	66024765	66035332	12	3	1058	2.5035	6.15E-03	0.678027
CACNG3	16	24266874	24373737	262	44	1058	2.5016	6.18E-03	0.678027
CD164	6	109687717	109703762	34	4	1058	2.4968	6.27E-03	0.678562
AMBP	9	116822407	116840752	37	7	1058	2.496	6.28E-03	0.678562
GRTP1	13	113978505	114018463	78	10	1058	2.4931	6.33E-03	0.678562
TAAR8	6	132873832	132874860	1	1	1058	2.4925	6.34E-03	0.678562
PDGFC	4	157682763	157892546	212	17	1058	2.4889	6.41E-03	0.678562
PSMD9	12	122326637	122355771	55	9	1058	2.4859	6.46E-03	0.678562
ADGRE1	19	6887560	6940464	231	18	1058	2.4835	6.50E-03	0.678562
CA1	8	86240458	86290342	62	10	1058	2.4783	6.60E-03	0.678562
SLC25A16	10	70242090	70287280	141	11	1058	2.4726	6.71E-03	0.678562

CCDC3	10	12938625	13141762	729	96	1058	2.4697	6.76E-03	0.678562
ESM1	5	54273695	54281414	13	2	1058	2.4683	6.79E-03	0.678562
ANXA2	15	60639350	60690185	80	11	1058	2.4676	6.80E-03	0.678562
DYNLL1	12	120907660	120936298	72	10	1058	2.4672	6.81E-03	0.678562
FITM1	14	24600675	24602058	2	1	1058	2.4658	6.84E-03	0.678562
TAS2R50	12	11138512	11139511	3	2	1058	2.4644	6.86E-03	0.678562
CST11	20	23431041	23433482	3	2	1058	2.4626	6.90E-03	0.678562
RNF44	5	175953700	175965026	5	2	1058	2.4616	6.92E-03	0.678562
PPP6R3	11	68228186	68382802	178	7	1058	2.4611	6.92E-03	0.678562
CHCHD5	2	113342023	113346617	10	3	1058	2.4609	6.93E-03	0.678562
ZNF850	19	37234380	37263717	63	8	1058	2.4591	6.96E-03	0.678562
ZNF567	19	37178466	37212428	63	5	1058	2.4546	7.05E-03	0.679622
CKMT2	5	80528605	80562217	73	7	1058	2.4501	7.14E-03	0.679622
RNF125	18	29598445	29653154	70	8	1058	2.447	7.20E-03	0.679622
DNAAF1	16	84178865	84211524	116	18	1058	2.4434	7.27E-03	0.679622
C15orf62	15	41062159	41064647	2	1	1057	2.4411	7.32E-03	0.679622
SOS2	14	50583846	50698596	268	19	1058	2.4399	7.35E-03	0.679622
ZNF704	8	81540686	81787016	213	10	1058	2.4397	7.35E-03	0.679622
BMP2	20	6748745	6760910	12	3	1058	2.4365	7.41E-03	0.679622
PPIL6	6	109711418	109762374	106	9	1058	2.4356	7.43E-03	0.679622
TAF6L	11	62538775	62554814	13	3	1058	2.4302	7.55E-03	0.679622
UFSP2	4	186320691	186347139	65	10	1058	2.4295	7.56E-03	0.679622
TIMP2	17	76849059	76921472	226	24	1058	2.4274	7.60E-03	0.679622
BCDIN3D	12	50229826	50236912	13	4	1058	2.427	7.61E-03	0.679622
ONECUT2	18	55102917	55158530	137	13	1058	2.4267	7.62E-03	0.679622
OR10K1	1	158435352	158436293	1	1	1058	2.4257	7.64E-03	0.679622
GPR146	7	1084218	1098905	79	8	1058	2.4235	7.69E-03	0.679622
FGFR1OP	6	167412805	167458064	224	9	1058	2.4203	7.75E-03	0.679622
11-Mar	5	16067474	16179897	130	13	1058	2.4194	7.77E-03	0.679622
ZSCAN1	19	58545434	58565999	16	2	1058	2.419	7.78E-03	0.679622
TAS2R7	12	10954131	10955226	1	1	1058	2.4166	7.83E-03	0.679622
MRPL18	6	160211492	160219461	35	4	1058	2.415	7.87E-03	0.679622
HOXB5	17	46668619	46671103	8	4	1058	2.4136	7.90E-03	0.679622
SMG5	1	156219015	156252676	66	7	1058	2.4132	7.91E-03	0.679622
FAM65A	16	67562717	67580691	17	4	1058	2.41	7.98E-03	0.679622

PRSS16	6	27215480	27224399	18	6	1058	2.4048	8.09E-03	0.679622
KIF2A	5	61601989	61683011	159	12	1058	2.4032	8.13E-03	0.679622
WDR11	10	122610687	122669038	185	9	1058	2.4031	8.13E-03	0.679622
CCRL2	3	46448721	46454488	15	3	1058	2.3986	8.23E-03	0.679622

Table S17. MAGMA Pathway-Test Results for SNP x AB42 GWAS for Hip. Slopes (subset to unadjusted $p < 0.05$)

FULL_NAME	NGENES	BETA	BETA_STD	SE	P	p.fdr
GO_HEMATOPOIETIC_PROGENITOR_CELL_DIFFERENTIATION	152	0.22024	0.020471	0.066132	0.000435	0.845575
GO_REGULATION_OF_DOUBLE_STRAND_BREAK_REPAIR	58	0.3268	0.018815	0.098706	0.000466	0.845575
GO_STAT_FAMILY_PROTEIN_BINDING	7	0.80422	0.016109	0.24481	0.000511	0.845575
GO_CENTRAL_ELEMENT	7	1.0629	0.02129	0.32526	0.000543	0.845575
REACTOME_DEGRADATION_OF_BETA_CATENIN_BY_THE_DESTRUCTION_COMPLEX	77	0.30598	0.020286	0.093733	0.00055	0.845575
GO_SCF_DEPENDENT_PROTEASOMAL_UBIQUITIN_DEPENDENT_PROTEIN_CATABOLIC_PROCESS	83	0.2806	0.019311	0.087299	0.000655	0.845575
GO_POSITIVE_REGULATION_OF_ARP2_3_COMPLEX_MEDIATED_ACTIN_NUCLEATION	7	0.9234	0.018496	0.29026	0.000734	0.845575
GO_PROTEIN_LOCALIZATION_TO_CELL_CORTEX	10	0.75214	0.018005	0.23928	0.000837	0.845575
REACTOME_NEF_MEDIATED_CD8_DOWN_REGULATION	7	0.79415	0.015907	0.25291	0.000846	0.845575
GO_CELLULAR_RESPONSE_TO_PROSTAGLANDIN_E_STIMULUS	15	0.73631	0.021585	0.23593	0.000903	0.845575
GO_NEGATIVE_REGULATION_OF_CELL_CYCLE_PHASE_TRANSITION	220	0.16786	0.018734	0.054026	0.000946	0.845575
REACTOME_TRANSCRIPTIONAL_REGULATION_BY_RUNX2	108	0.24359	0.01911	0.079302	0.001066	0.845575
GO_POSITIVE_REGULATION_OF_ACTIN_NUCLEATION	16	0.59016	0.017867	0.19278	0.001104	0.845575
GO_RESPONSE_TO_PROSTAGLANDIN_E	20	0.59595	0.02017	0.19674	0.001229	0.845575
GO_TUBE_CLOSURE	85	0.26895	0.018731	0.088988	0.001256	0.845575
GO_CELLULAR_RESPONSE_TO_PROSTAGLANDIN_STIMULUS	21	0.60684	0.021045	0.2011	0.001276	0.845575
GO_PROTEIN_KINASE_ACTIVITY	536	0.1009	0.017416	0.033567	0.001326	0.845575
BIOCARTA_AHSP_PATHWAY	9	0.66923	0.015199	0.2227	0.00133	0.845575
GO_MRE11_COMPLEX	5	0.89506	0.015153	0.29943	0.001401	0.845575
GO_2_ACYLGLYCEROL_O_ACYLTRANSFERASE_ACTIVITY	5	1.1969	0.020263	0.40242	0.001471	0.845575
GO_PROTEIN_SERINE_THREONINE_KINASE_ACTIVITY	391	0.11398	0.016873	0.03861	0.001581	0.845575
REACTOME_CROSS_PRESENTATION_OF_PARTICULATE_EXOGENOUS_ANTIGENS_PHAGOSOMES	6	0.89152	0.016533	0.30299	0.001631	0.845575
REACTOME_NEF_MEDIATED_CD4_DOWN_REGULATION	9	0.6271	0.014242	0.21419	0.00171	0.845575
REACTOME_RETROGRADE_NEUROTROPHIN_SIGNALLING	14	0.57996	0.016425	0.19853	0.001745	0.845575
REACTOME_GAP_JUNCTION_TRAFFICKING_AND_REGULATION	39	0.38006	0.017952	0.13056	0.001804	0.845575
GO_CALCIUM_CATION_ANTIPORTER_ACTIVITY	9	0.85541	0.019427	0.29694	0.001987	0.845575
GO_MITOTIC_DNA_INTEGRITY_CHECKPOINT	98	0.21999	0.016444	0.076504	0.00202	0.845575
REACTOME_SCF_SKP2_MEDIATED_DEGRADATION_OF_P27_P21	56	0.29029	0.016423	0.10272	0.002359	0.845575
GO_ATP_TRANSMEMBRANE_TRANSPORTER_ACTIVITY	8	0.75413	0.016148	0.26745	0.002406	0.845575
GO_PHOSPHORUS_OXYGEN_LYASE_ACTIVITY	18	0.53352	0.017131	0.1898	0.002473	0.845575
GO_REGULATION_OF_STEM_CELL_DIFFERENTIATION	105	0.21631	0.016734	0.07696	0.002475	0.845575
GO_NEURON_PROJECTION_MAINTENANCE	9	0.64119	0.014562	0.2287	0.00253	0.845575
GO_INNATE_IMMUNE_RESPONSE_IN_MUCOSA	17	0.53994	0.016849	0.19329	0.002611	0.845575
REACTOME_HEME_BIOSYNTHESIS	10	0.64196	0.015368	0.22982	0.002612	0.845575
REACTOME_NEF_MEDIATES_DOWN_MODULATION_OF_CELL_SURFACE_RECEPTORS_BY_RECRUITING_THEM_TO_CLATHRIN_ADAPTERS	20	0.40439	0.013686	0.14524	0.002686	0.845575
GO_RESPONSE_TO_PROSTAGLANDIN	28	0.46859	0.018761	0.16896	0.002778	0.845575
REACTOME_CYCLIN_A:CDK2_ASSOCIATED_EVENTS_AT_S_PHASE_ENTRY	81	0.22591	0.01536	0.081556	0.002806	0.845575
GO_RECEPTOR_GUANYLYL_CYCLASE_SIGNALING_PATHWAY	9	0.62066	0.014096	0.22471	0.002875	0.845575
GO_ACTIVATION_OF_JNKK_ACTIVITY	10	0.66128	0.01583	0.23955	0.002889	0.845575
GO_RETINAL_BIPOLAR_NEURON_DIFFERENTIATION	5	1.0574	0.017901	0.38436	0.002974	0.845575
GO_INSULIN_RECEPTOR_COMPLEX	5	1.1613	0.019661	0.42259	0.003001	0.845575
GO_SCAR_COMPLEX	8	0.83541	0.017888	0.30451	0.003044	0.845575
REACTOME_TCF_DEPENDENT_SIGNALING_IN_RESPONSE_TO_WNT	197	0.15571	0.016456	0.056921	0.003117	0.845575
GO_PEROXISOME_TARGETING_SEQUENCE_BINDING	4	0.98719	0.014949	0.36131	0.003149	0.845575
GO_PIRNA_BIOSYNTHETIC_PROCESS	5	0.85526	0.014479	0.3131	0.003155	0.845575
GO_REGULATION_OF_DEFENSE_RESPONSE_TO_VIRUS_BY_VIRUS	28	0.35297	0.014132	0.12973	0.00326	0.845575
GO_OPSONIZATION	11	0.73146	0.018364	0.26927	0.003303	0.845575
GO_POSITIVE_REGULATION_OF_VIRAL_ENTRY_INTO_HOST_CELL	9	0.62106	0.014105	0.22914	0.003364	0.845575

GO_PROTEIN_POLYUBIQUITINATION	279	0.12999	0.01631	0.048212	0.00351	0.845575
GO_VITAMIN_A_METABOLIC_PROCESS	7	0.75037	0.01503	0.27955	0.003639	0.845575
GO_ORGAN_OR_TISSUE_SPECIFIC_IMMUNE_RESPONSE	31	0.37337	0.015727	0.13931	0.003684	0.845575
REACTOME_METABOLISM_OF_PORPHYRINS	15	0.51938	0.015225	0.19386	0.003694	0.845575
GO_PROTEASOME_REGULATORY_PARTICLE_BASE_SUBCOMPLEX	11	0.69253	0.017387	0.25858	0.003705	0.845575
GO_STEROID_HORMONE_BINDING	6	0.7946	0.014736	0.29715	0.003751	0.845575
REACTOME_NEGATIVE_REGULATION_OF_NOTCH4_SIGNALING	50	0.31062	0.016608	0.11663	0.003875	0.846261
GO_MODIFICATION_DEPENDENT_PROTEIN_BINDING	117	0.18995	0.015506	0.07148	0.003942	0.846261
GO_NEGATIVE_REGULATION_OF_PROTEIN_LOCALIZATION_TO_CELL_PERIPHERY	24	0.36621	0.013576	0.13796	0.003977	0.846261
REACTOME_REGULATION_OF_RUNX2_EXPRESSION_AND_ACTIVITY	69	0.26604	0.016701	0.1004	0.004032	0.846261
PID_INTEGRIN5_PATHWAY	15	0.53198	0.015595	0.20249	0.004308	0.858227
GO_MITOCHONDRIAL_RESPIRATORY_CHAIN_COMPLEX_III_ASSEMBLY	9	0.60341	0.013704	0.22991	0.004343	0.858227
GO_GOLGI_RIBBON_FORMATION	12	0.60138	0.01577	0.23245	0.004843	0.858227
GO_NEGATIVE_REGULATION_OF_PROTEIN_LOCALIZATION_TO_MEMBRANE	28	0.33585	0.013446	0.12982	0.004844	0.858227
GO_NEGATIVE_REGULATION_OF_MITOTIC_CELL_CYCLE	285	0.12077	0.015313	0.046771	0.004912	0.858227
BIOCARTA_FBW7_PATHWAY	9	0.61578	0.013985	0.23979	0.005119	0.858227
REACTOME_DISEASES_ASSOCIATED_WITH_VISUAL_TRANSDUCTION	11	0.61032	0.015323	0.23856	0.005264	0.858227
GO_KINASE_ACTIVITY	683	0.076598	0.014859	0.029945	0.00527	0.858227
GO_RESPONSE_TO_ACTIVITY	58	0.2776	0.015982	0.10869	0.005329	0.858227
REACTOME_RHO_GTPASES_ACTIVATE_KTN1	11	0.6173	0.015498	0.242	0.005379	0.858227
GO_OXIDOREDUCTASE_ACTIVITY_OXIDIZING_METAL_IONS	14	0.47734	0.013519	0.18725	0.005404	0.858227
REACTOME_DEGRADATION_OF_GLI1_BY_THE_PROTEASOME	56	0.27832	0.015746	0.10923	0.005421	0.858227
GO_ANTIGEN_PROCESSING_AND_PRESENTATION_OF_EXOGENOUS_PEPTIDE_ANTIGEN_VIA_MHC_CLASS_I	70	0.23956	0.015147	0.094059	0.005438	0.858227
GO_PROTOPORPHYRINOGEN_IX_BIOSYNTHETIC_PROCESS	8	0.60191	0.012889	0.23658	0.005481	0.858227
GO_ENDODERM_FORMATION	48	0.31449	0.016476	0.12376	0.005531	0.858227
GO_REGULATION_OF_PRESYNAPTIC_MEMBRANE_POTENTIAL	7	0.74957	0.015014	0.29567	0.005625	0.858227
GO_ATP_HYDROLYSIS_COUPLED_TRANSMEMBRANE_TRANSPORT	28	0.38536	0.015429	0.15214	0.005661	0.858227
GO_RESPONSE_TO_DEFENSES_OF_OTHER_ORGANISM_INVOLVED_IN_SYMBIOTIC_INTERACTION	5	0.92351	0.015635	0.36497	0.005702	0.858227
GO_SULFUR_AMINO_ACID_METABOLIC_PROCESS	16	0.48698	0.014744	0.19293	0.005804	0.858227
GO_METHIONINE_METABOLIC_PROCESS	6	0.76625	0.01421	0.30367	0.005817	0.858227
GO_REGULATION_OF_TRANSPOSITION	20	0.45624	0.015441	0.18087	0.005832	0.858227
GO_CELLULAR_RESPONSE_TO_INSULIN_LIKE_GROWTH_FACTOR_STIMULUS	8	0.61966	0.013269	0.24617	0.00592	0.858227
GO_STEM_CELL_DIFFERENTIATION	234	0.13568	0.015611	0.053904	0.00592	0.858227
GO_CYCLASE_ACTIVITY	17	0.48333	0.015083	0.19295	0.006129	0.858227
GO_PHOTORECEPTOR_CELL_DEVELOPMENT	44	0.30525	0.015313	0.12189	0.006139	0.858227
GO_REGULATION_OF_DOPAMINERGIC_NEURON_DIFFERENTIATION	9	0.63943	0.014522	0.25581	0.006221	0.858227
GO_MAP_KINASE_KINASE_KINASE_ACTIVITY	24	0.38086	0.014119	0.15309	0.006432	0.858227
GO_LONG_CHAIN_FATTY_ACYL_COA_BINDING	6	0.68603	0.012722	0.27638	0.006534	0.858227
GO_PROTEIN_SULFATION	8	0.76654	0.016414	0.31024	0.006746	0.858227
REACTOME_RHO_GTPASE_EFFECTORS	274	0.11916	0.014818	0.048355	0.006869	0.858227
GO_NEURAL_TUBE_FORMATION	98	0.20251	0.015138	0.082219	0.006894	0.858227
GO_ESTABLISHMENT_OF_MEIOTIC_SPINDLE_LOCALIZATION	5	0.7467	0.012641	0.30317	0.006895	0.858227
GO_CILIUM_OR_FLAGELLUM_DEPENDENT_CELL_MOTILITY	35	0.33098	0.014812	0.13446	0.006923	0.858227
REACTOME_CREB3_FACTORS_ACTIVATE_GENES	8	0.71555	0.015322	0.29104	0.006979	0.858227
GO_JUN_KINASE_KINASE_KINASE_ACTIVITY	8	0.62919	0.013473	0.25594	0.006984	0.858227
GO_HOMOCYSTEINE_METABOLIC_PROCESS	11	0.53386	0.013403	0.21781	0.007127	0.858227
GO_REGULATION_OF_RESTING_MEMBRANE_POTENTIAL	8	0.66549	0.01425	0.27195	0.007206	0.858227
GO_ACTIVATION_OF_JUN_KINASE_ACTIVITY	35	0.31176	0.013952	0.12743	0.007217	0.858227
GO_CYCLIC_NUCLEOTIDE_BIOSYNTHETIC_PROCESS	18	0.45913	0.014743	0.18785	0.007264	0.858227
GO_CELLULAR_SODIUM_ION_HOMEOSTASIS	16	0.49372	0.014948	0.20213	0.007296	0.858227
REACTOME_SYNDECAN_INTERACTIONS	26	0.3798	0.014654	0.15583	0.007405	0.858227
GO_ENERGY_COUPLED_PROTON_TRANSMEMBRANE_TRANSPORT_AGAINST_ELECTROCHEMICAL_GRADIENT	26	0.37412	0.014435	0.15361	0.007441	0.858227
GO_RETINOL_BINDING	14	0.46357	0.013129	0.1905	0.007485	0.858227
GO_MONONUCLEAR_CELL_DIFFERENTIATION	33	0.34626	0.015048	0.14247	0.007546	0.858227
REACTOME_FCERI_MEDIATED_NF_KB_ACTIVATION	74	0.23229	0.015099	0.095611	0.007565	0.858227

GO_MICROVESICLE	3	1.1665	0.015299	0.4805	0.007601	0.858227
GO_COMPLEMENT_RECEPTOR_MEDIATED_SIGNALING_PATHWAY	10	0.6893	0.016501	0.2851	0.007814	0.858227
PID_AR_PATHWAY	57	0.2512	0.014337	0.1041	0.007916	0.858227
GO_HOMOSERINE_METABOLIC_PROCESS	4	0.94177	0.014261	0.39147	0.008077	0.858227
GO_MECHANOSENSORY_BEHAVIOR	15	0.48309	0.014162	0.20114	0.008164	0.858227
GO_HEMATOPOIETIC_STEM_CELL_DIFFERENTIATION	74	0.22052	0.014334	0.091822	0.008168	0.858227
GO_PROTEIN_PRENYLTRANSFERASE_ACTIVITY	6	0.74823	0.013876	0.31182	0.008214	0.858227
GO_SODIUM_ION_EXPORT_ACROSS_PLASMA_MEMBRANE	14	0.52078	0.014749	0.21717	0.008247	0.858227
GO_PHOTOTRANSDUCTION	50	0.24261	0.012972	0.10118	0.00825	0.858227
REACTOME_MITOCHONDRIAL_FATTY_ACID_BETA_OXIDATION_OF_UNSATURATED_FATTY_ACIDS	6	0.60073	0.011141	0.25057	0.00826	0.858227
GO_OXIDOREDUCTASE_ACTIVITY_ACTING_ON_THE_CH_CH_GROUP_OF_DONORS	57	0.24732	0.014116	0.10321	0.008286	0.858227
REACTOME_CELLULAR_RESPONSES_TO_EXTERNAL_STIMULI	472	0.08636	0.014014	0.036052	0.008307	0.858227
BIOCARTA_SKP2E2F_PATHWAY	10	0.55355	0.013251	0.23122	0.008337	0.858227
GO_WIDE_PORE_CHANNEL_ACTIVITY	17	0.47523	0.01483	0.19868	0.008384	0.858227
REACTOME_THE_ROLE_OF_NEF_IN_HIV_1_REPLICATION_AND_DISEASE_PATHOGENESIS	27	0.31593	0.012421	0.13211	0.008399	0.858227
GO_POSITIVE_REGULATION_OF_MICROGLIAL_CELL_MIGRATION	5	0.72583	0.012288	0.30399	0.008484	0.858227
GO_UBIQUITIN_CONJUGATING_ENZYME_BINDING	30	0.34867	0.014449	0.14606	0.008495	0.858227
GO_NUCLEOTIDE_TRANSMEMBRANE_TRANSPORTER_ACTIVITY	16	0.4296	0.013006	0.18007	0.00853	0.858227
GO_POSITIVE_REGULATION_OF_CHROMATIN_BINDING	12	0.5328	0.013971	0.22451	0.008824	0.875595
GO_ASTROCYTE_CELL_MIGRATION	8	0.65265	0.013975	0.27525	0.008872	0.875595
GO_MRNA_3_END_PROCESSING	91	0.19053	0.013727	0.08042	0.008919	0.875595
GO_BRAIN_DERIVED_NEUROTROPHIC_FACTOR_RECEPTOR_SIGNALING_PATHWAY	5	0.95055	0.016093	0.4027	0.009133	0.889452
GO_NEURAL_PLATE_PATTERN_SPECIFICATION	7	0.74438	0.01491	0.3166	0.009364	0.901003
GO_ATP_TRANSPORT	9	0.58303	0.013241	0.24824	0.009426	0.901003
GO_NEGATIVE_REGULATION_OF_CELL_CYCLE_PROCESS	301	0.10765	0.014019	0.045982	0.00962	0.901003
GO_PROTEIN_GERANYLGERANYLTRANSFERASE_ACTIVITY	4	0.92351	0.013984	0.39538	0.009759	0.901003
GO_PLASMA_LIPOPROTEIN_PARTICLE_CLEARANCE	52	0.25772	0.014051	0.11053	0.009865	0.901003
GO_POSITIVE_REGULATION_OF_PROTEIN_BINDING	86	0.18313	0.012828	0.079313	0.010479	0.901003
GO_SCF_UBIQUITIN_LIGASE_COMPLEX	56	0.23562	0.01333	0.1023	0.010637	0.901003
REACTOME_SYNTHESIS_OF_PG	8	0.61006	0.013063	0.26489	0.010645	0.901003
REACTOME_SWITCHING_OF_ORIGINS_TO_A_POST_REPLICATIVE_STATE	83	0.20431	0.014061	0.088761	0.01068	0.901003
GO_GAP_JUNCTION_CHANNEL_ACTIVITY	10	0.62132	0.014874	0.26996	0.010688	0.901003
REACTOME_FBXW7_MUTANTS_AND_NOTCH1_IN_CANCER	5	0.87556	0.014823	0.38119	0.010818	0.901003
GO_GLYCOSYL_COMPOUND_CATABOLIC_PROCESS	37	0.31357	0.014428	0.13655	0.010832	0.901003
GO_INOSITOL_PHOSPHATE_CATABOLIC_PROCESS	15	0.41476	0.012159	0.18066	0.010849	0.901003
GO_RESPONSE_TO_AUDITORY_STIMULUS	22	0.35351	0.012548	0.15414	0.010917	0.901003
GO_CALCIUM_SODIUM_ANTIPORTER_ACTIVITY	5	0.81756	0.013841	0.35651	0.010925	0.901003
REACTOME_CDK_MEDIATED_PHOSPHORYLATION_AND_REMOVAL_OF_CDC6	66	0.2216	0.013607	0.096992	0.011171	0.901003
REACTOME_CELLULAR_RESPONSES_TO_STRESS	385	0.089411	0.013137	0.039228	0.011332	0.901003
GO_RESPONSE_TO_ESTRADIOL	117	0.16057	0.013107	0.070528	0.011411	0.901003
GO_REGULATION_OF_PROTEIN_LOCALIZATION_TO_CELL_PERIPHERY	103	0.16744	0.01283	0.073598	0.011457	0.901003
GO_PHOSPHOLIPASE_A1_ACTIVITY	4	1.1305	0.017118	0.49754	0.011547	0.901003
REACTOME_FORMATION_OF_THE_BETA_CATENIN:TCF_TRANSACTIVATING_COMPLEX	68	0.22978	0.01432	0.10128	0.011649	0.901003
GO_RNA_DIRECTED_DNA_POLYMERASE_ACTIVITY	5	0.63632	0.010773	0.28048	0.011649	0.901003
GO_TELOMERASE_ACTIVITY	5	0.63632	0.010773	0.28048	0.011649	0.901003
GO_RESPONSE_TO_IMMUNE_RESPONSE_OF_OTHER_ORGANISM_INVOLVED_IN_SYMBIOTIC_INTERACTION	3	1.1187	0.014671	0.49324	0.011671	0.901003
GO_REGULATION_OF_PROTEIN_LOCALIZATION_TO_PLASMA_MEMBRANE	87	0.1791	0.012618	0.078975	0.011676	0.901003
REACTOME_DEGRADATION_OF_DVL	53	0.2476	0.013628	0.10931	0.011763	0.901003
REACTOME_REGULATION_OF_PTEN_STABILITY_AND_ACTIVITY	64	0.23314	0.014097	0.10311	0.011882	0.901003
GO_SULFUR_AMINO_ACID_CATABOLIC_PROCESS	9	0.56707	0.012879	0.25148	0.012077	0.901003
GO_NEGATIVE_REGULATION_OF_EXTRACELLULAR_MATRIX_ORGANIZATION	10	0.63759	0.015263	0.28325	0.0122	0.901003
GO_LOW_DENSITY_LIPOPROTEIN_PARTICLE_CLEARANCE	34	0.30196	0.01332	0.13436	0.012312	0.901003
REACTOME_TYSND1_CLEAVES_PEROXISOMAL_PROTEINS	7	0.60931	0.012205	0.27116	0.012326	0.901003
GO_POSITIVE_REGULATION_OF_DOUBLE_STRAND_BREAK_REPAIR_VIA_NONHOMOLOGOUS_END_JOINING	8	0.61978	0.013271	0.27591	0.012348	0.901003
GO_POSITIVE_REGULATION_OF_DOUBLE_STRAND_BREAK_REPAIR	22	0.37763	0.013404	0.16813	0.012357	0.901003

REACTOME_SODIUM_CALCIUM_EXCHANGERS	10	0.69059	0.016532	0.30763	0.012396	0.901003
GO_CELLULAR_MODIFIED_AMINO_ACID_CATABOLIC_PROCESS	23	0.37046	0.013445	0.16514	0.012443	0.901003
KEGG_VASOPRESSIN_REGULATED_WATER_REABSORPTION	39	0.29687	0.014023	0.13253	0.01255	0.901003
GO_CANONICAL_WNT_SIGNALING_PATHWAY_INVOLVED_IN_REGULATION_OF_CELL_PROLIFERATION	6	0.59052	0.010951	0.26402	0.012663	0.901003
REACTOME_MITOTIC_METAPHASE_AND_ANAPHASE	178	0.13261	0.013328	0.059393	0.012791	0.901003
GO_OSTEOBLAST_PROLIFERATION	22	0.39127	0.013888	0.17537	0.012844	0.901003
GO_AMINO_SUGAR_METABOLIC_PROCESS	20	0.42051	0.014232	0.18853	0.012865	0.901003
GO_G1_DNA_DAMAGE_CHECKPOINT	61	0.2221	0.013112	0.099689	0.012951	0.901003
GO_ADP_TRANSPORT	4	0.77279	0.011702	0.34761	0.013109	0.901003
GO_ADP_TRANSMEMBRANE_TRANSPORTER_ACTIVITY	4	0.77279	0.011702	0.34761	0.013109	0.901003
GO_FIBROBLAST_PROLIFERATION	74	0.21618	0.014052	0.097276	0.013137	0.901003
REACTOME_PINK_PARKIN_MEDIATED_MITOPHAGY	19	0.38258	0.012621	0.17219	0.013155	0.901003
GO_NEGATIVE_REGULATION_OF_CELL_ADHESION_MEDIATED_BY_INTEGRIN	10	0.53146	0.012723	0.23931	0.01319	0.901003
REACTOME_S_PHASE	149	0.14169	0.013041	0.063876	0.013276	0.901003
GO_MITOTIC_CELL_CYCLE_CHECKPOINT	154	0.13862	0.012968	0.0625	0.013288	0.901003
GO_PROTEIN_PHOSPHORYLATION	1729	0.043526	0.013008	0.019629	0.013305	0.901003
GO_REGULATION_OF_EXTRACELLULAR_MATRIX_ORGANIZATION	35	0.30706	0.013742	0.13857	0.013356	0.901003
GO_EXTRACELLULAR_MATRIX_ASSEMBLY	22	0.37607	0.013349	0.16997	0.01347	0.901003
BIOCARTA_P27_PATHWAY	13	0.43949	0.011995	0.19871	0.013499	0.901003
GO_PHOSPHATIDYLINOSITOL_BIOSYNTHETIC_PROCESS	104	0.16576	0.012762	0.074948	0.013502	0.901003
GO_SOMITE_SPECIFICATION	3	1.221	0.016012	0.55274	0.013596	0.901003
REACTOME_NON_INTEGRIN_MEMBRANE_ECM_INTERACTIONS	53	0.2524	0.013893	0.11435	0.013658	0.901003
PID_UPA_UPAR_PATHWAY	41	0.29689	0.014378	0.13459	0.013703	0.901003
REACTOME_DNA_DAMAGE_TELOMERE_STRESS_INDUCED_SENESCENCE	62	0.20959	0.012474	0.095193	0.01385	0.901003
GO_IMMUNOGLOBULIN_RECEPTOR_BINDING	4	0.97078	0.0147	0.44097	0.01386	0.901003
PID_BETA_CATENIN_DEG_PATHWAY	16	0.41268	0.012494	0.1876	0.013918	0.901003
GO_OXIDOREDUCTASE_ACTIVITY_ACTING_ON_OTHER_NITROGENOUS_COMPOUNDS_AS_DONORS	5	0.9438	0.015978	0.42935	0.013975	0.901003
REACTOME_RUNX1_REGULATES_TRANSCRIPTION_OF_GENES_INVOLVED_IN_DIFFERENTIATION_OF_HSCS	103	0.17362	0.013304	0.079001	0.013991	0.901003
GO_SEMAPHORIN_PLEXIN_SIGNALING_PATHWAY	35	0.32564	0.014574	0.1482	0.014004	0.901003
GO_PROTEIN_AUTOPHOSPHORYLATION	215	0.11448	0.012632	0.05212	0.014038	0.901003
GO_CELLULAR_POTASSIUM_ION_HOMEOSTASIS	13	0.50045	0.013658	0.22804	0.014104	0.901003
REACTOME_MITOPHAGY	25	0.32611	0.012338	0.14869	0.014154	0.901003
GO_POSTSYNAPTIC_SPECIALIZATION_ASSEMBLY	21	0.40675	0.014106	0.18553	0.014183	0.901003
GO_SMN_COMPLEX	8	0.496	0.010621	0.22632	0.01421	0.901003
REACTOME_GAP_JUNCTION_ASSEMBLY	26	0.35913	0.013856	0.1643	0.014424	0.909833
REACTOME_ACTIVATED_NTRK2_SIGNALS_THROUGH_CDK5	6	0.66193	0.012276	0.30351	0.014602	0.912679
REACTOME_RECYCLING_PATHWAY_OF_L1	37	0.27995	0.012881	0.12846	0.014665	0.912679
REACTOME_ORC1_REMOVAL_FROM_CHROMATIN	66	0.21989	0.013501	0.101	0.014744	0.912679
GO_PHOSPHOLIPID_METABOLIC_PROCESS	220	0.11538	0.012877	0.053013	0.014769	0.912679
GO_PURINE_NUCLEOTIDE_TRANSMEMBRANE_TRANSPORTER_ACTIVITY	12	0.46541	0.012204	0.21406	0.014851	0.913112
GO_TRANS_SYNAPTIC_SIGNALING_MODULATING_SYNAPTIC_TRANSMISSION	10	0.5696	0.013635	0.26269	0.015074	0.918007
REACTOME_CELLULAR_RESPONSE_TO_HYPOXIA	69	0.20696	0.012992	0.095498	0.01512	0.918007
REACTOME_NUCLEAR_ENVELOPE_REASSEMBLY	12	0.4523	0.01186	0.20888	0.015187	0.918007
GO_PEPTIDASE_COMPLEX	81	0.17993	0.012234	0.083218	0.015311	0.918007
GO_REGULATION_OF_HEMATOPOIETIC_PROGENITOR_CELL_DIFFERENTIATION	77	0.1925	0.012763	0.089143	0.015416	0.918007
GO_CYTOCHROME_COMPLEX_ASSEMBLY	32	0.2606	0.011153	0.12089	0.015559	0.918007
GO_REGULATION_OF_DENDRITE_MORPHOGENESIS	77	0.20712	0.013732	0.096081	0.015563	0.918007
GO_RECEPTOR_CATABOLIC_PROCESS	31	0.28406	0.011966	0.13222	0.015847	0.918007
GO_ISG15_PROTEIN_CONJUGATION	5	0.85669	0.014503	0.39921	0.015947	0.918007

Supplemental Figures

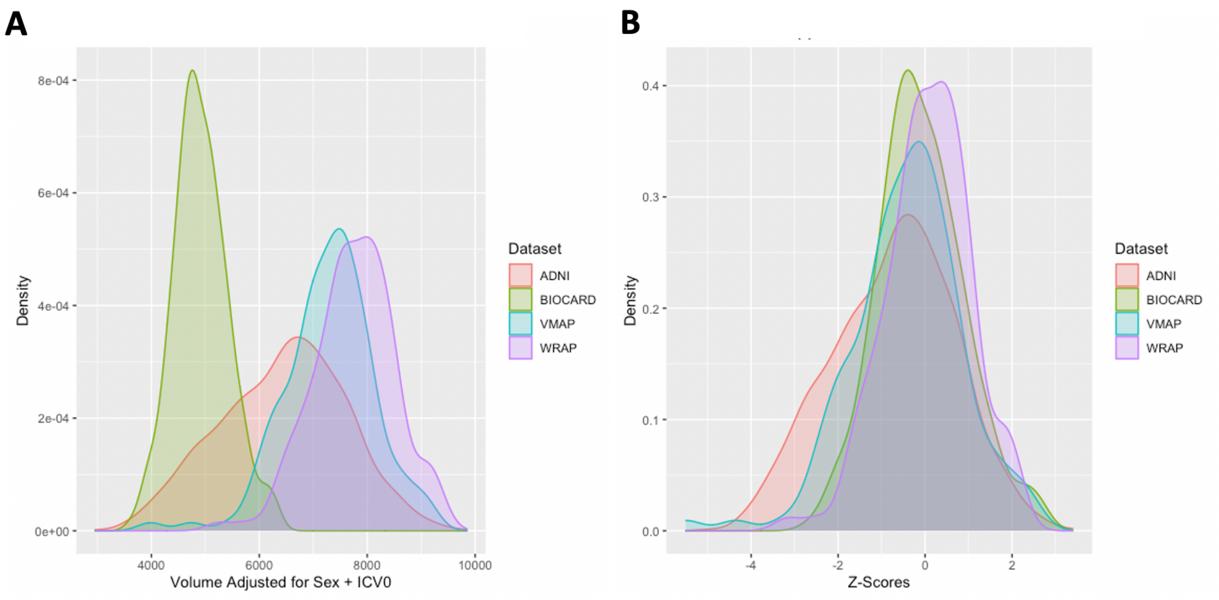

Supplemental Figure 1. Density plots of hippocampal volume distribution pre- and post-standardization per study. The plots are colored by dataset for visualization. The density is presented on the y-axis. **A)** Pre-standardized distribution of hippocampal volumes adjusted for sex and intracranial volume (in mm^3). **B)** Distribution of hippocampal volume Z-scores after standardization. All measures were centered and standardized using the mean and standard deviation of adjusted hippocampal volumes of cognitively normal participants.

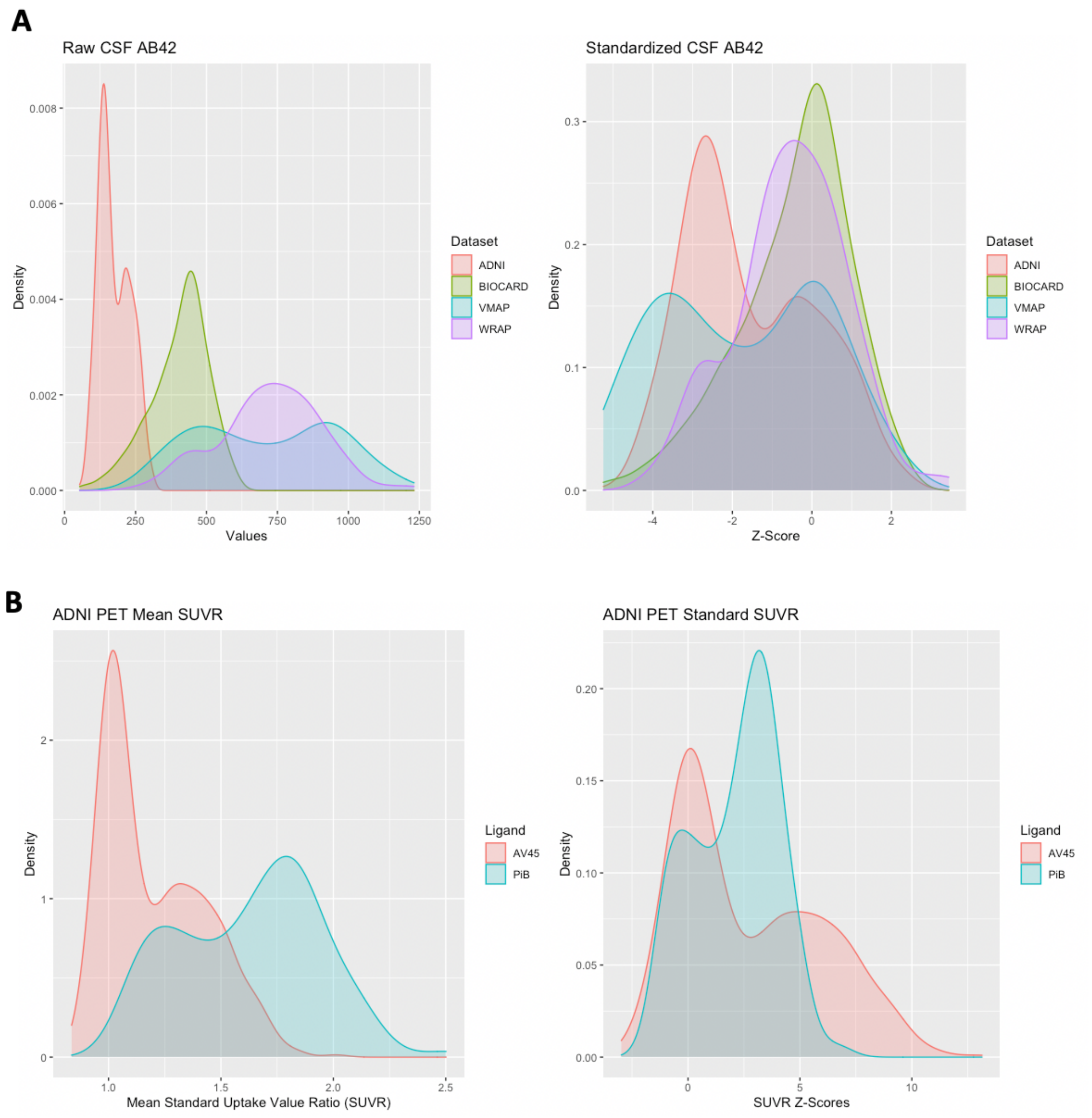

Supplementary Figure 2. Density plots of normalized amyloid measures. All measures were centered and standardized using the mean and standard deviation of the amyloid negative gaussian distribution within study from a mixture model. **A)** Density plot of **r**aw CSF Aβ-42 values colored by dataset (left). Density plot after harmonization (right), where the x-axis represents Z-scores. **B**) Left and right panels represent amyloid PET standard uptake value ratios pre- and post- harmonization. These density plots are colored by ligand.

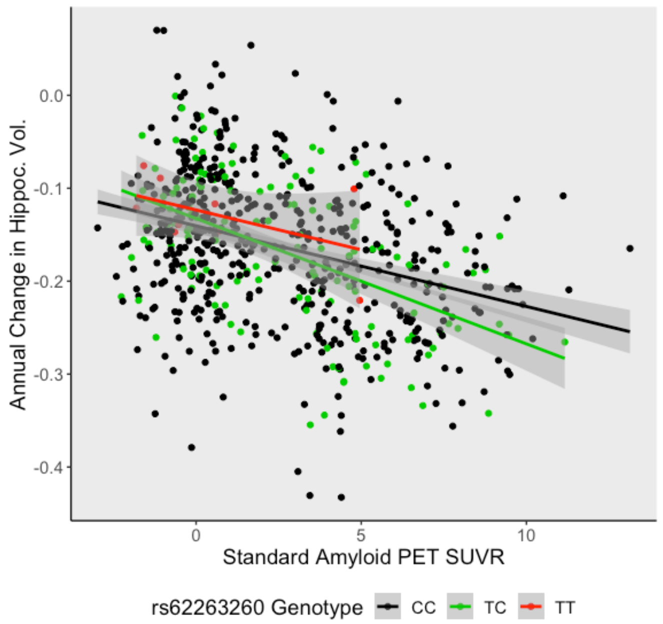

Supplemental Figure 3. A plot demonstrating how the index SNP, rs62263260, modifies the association between standardized baseline PET amyloid levels and hippocampal atrophy in ADNI. The y-axis represents baseline standardized hippocampal volume, and the x-axis represents standardized amyloid PET SUVRs of Aβ-42. Points and lines are color coded by genotype, where rs62263260-T heterozygotes are denoted by the green line and homozygotes (TT) are red.

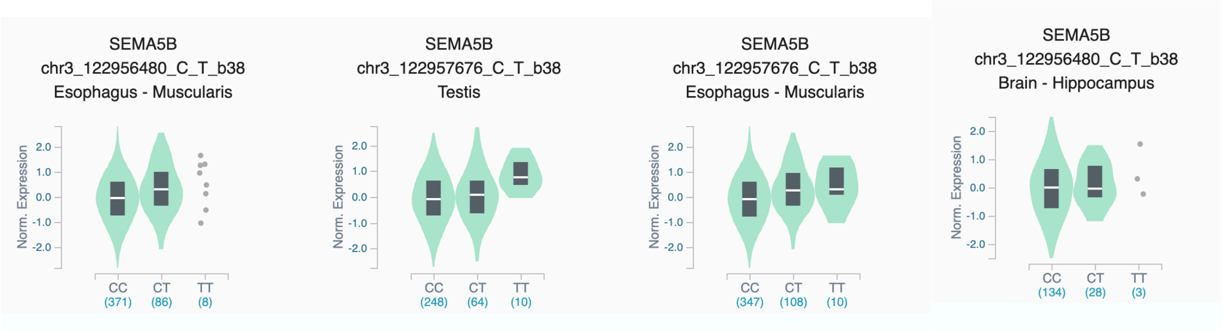

Supplemental Figure 4. Violin plots adapted from the NIH Gene-Tissue Expression (GTEx) Project showing the normalized expression of *SEMA5B* in esophageal, testicular, and brain tissue. The plots suggest that the minor alleles of rs62263260 and rs10934626, a SNP in the same locus, are associated with an increase in Semaphorin 5B expression, especially in homozygotes. These images were obtained from the GTEx Portal on 06/15/20. In addition, the given rs62263260 *SEMA5B* eQTL beta from Sieberts et al., 2020 is β= 0.318. The expression increasing allele is rs62263260-T, the minor allele.[18]

References

1. Jefferson AL, Gifford KA, Acosta LMY, Bell SP, Donahue MJ, Taylor Davis L *et al.* The Vanderbilt Memory & Aging Project: Study Design and Baseline Cohort Overview. *Journal of Alzheimer's Disease* 2016; (Preprint)**:** 1-20.

2. Johnson SC, Koscik RL, Jonaitis EM, Clark LR, Mueller KD, Berman SE *et al.* The Wisconsin Registry for Alzheimer's Prevention: A review of findings and current directions. *Alzheimer & Dementia: DADM* 2018; **10:** 130-142.

3. Moghekar A, Li S, Lu Y, Li M, Wang M-C, Albert M *et al.* CSF biomarker changes precede symptom onset of mild cognitive impairment. *Neurology* 2013; **81**(20)**:** 1753-1758.

4. Roberts RO, Geda YE, Knopman DS, Cha RH, Pankratz VS, Boeve BF *et al.* The Mayo Clinic Study of Aging: design and sampling, participation, baseline measures and sample characteristics. *Neuroepidemiology* 2008; **30**(1)**:** 58-69.

5. Ramanan VK, Wang X, Przybelski SA, Raghavan S, Heckman MG, Batzler A *et al.* Variants in PPP2R2B and IGF2BP3 are associated with higher tau deposition. *Brain Communications* 2020; **2**(2).

6. Ruigrok ANV, Salimi-Khorshidi G, Lai M-C, Baron-Cohen S, Lombardo MV, Tait RJ *et al.* A meta-analysis of sex differences in human brain structure. *Neuroscience & Biobehavioral Reviews* 2014; **39:** 34-50.

7. Ramanan VK, Lesnick TG, Przybelski SA, Heckman MG, Knopman DS, Graff-Radford J *et al.* Coping with brain amyloid: genetic heterogeneity and cognitive resilience to Alzheimer's pathophysiology. *Acta Neuropathol Commun* 2021; **9**(1)**:** 48.

8. Ashburner M, Ball CA, Blake JA, Botstein D, Butler H, Cherry JM *et al.* Gene ontology: tool for the unification of biology. The Gene Ontology Consortium. *Nature genetics* 2000; **25**(1)**:** 25-29.

9. Kanehisa M, Furumichi M, Tanabe M, Sato Y, Morishima K. KEGG: new perspectives on genomes, pathways, diseases and drugs. *Nucleic Acids Research* 2017; **45**(D1)**:** D353-D361.

10. Kanehisa M, Sato Y, Kawashima M, Furumichi M, Tanabe M. KEGG as a reference resource for gene and protein annotation. *Nucleic acids research* 2016; **44**(D1)**:** D457-D462.

11. Ogata H, Goto S, Sato K, Fujibuchi W, Bono H, Kanehisa M. KEGG: Kyoto Encyclopedia of Genes and Genomes. *Nucleic acids research* 1999; **27**(1)**:** 29-34.

12. Croft D, O'Kelly G, Wu G, Haw R, Gillespie M, Matthews L *et al.* Reactome: a database of reactions, pathways and biological processes. *Nucleic acids research* 2011; **39**(Database issue)**:** D691-D697.

13. Fabregat A, Sidiropoulos K, Garapati P, Gillespie M, Hausmann K, Haw R *et al.* The Reactome pathway Knowledgebase. *Nucleic acids research* 2016; **44**(D1)**:** D481-D487.

14. Nishimura D. BioCarta. *Biotech Software & Internet Report* 2001; **2**(3)**:** 117-120.

15. Schaefer CF, Anthony K, Krupa S, Buchoff J, Day M, Hannay T *et al.* PID: the Pathway Interaction Database. *Nucleic acids research* 2009; **37**(Database issue)**:** D674-D679.

16. Hunt JFV, Buckingham W, Kim AJ, Oh J, Vogt NM, Jonaitis EM *et al.* Association of Neighborhood-Level Disadvantage With Cerebral and Hippocampal Volume. *JAMA Neurol* 2020; **77**(4)**:** 451-460.

17. Pettigrew C, Soldan A, Zhu Y, Wang M-C, Brown T, Miller M *et al.* Cognitive reserve and cortical thickness in preclinical Alzheimer's disease. *Brain Imaging Behav* 2017; **11**(2)**:** 357-367.

18. Sieberts SK, Perumal TM, Carrasquillo MM, Allen M, Reddy JS, Hoffman GE *et al.* Large eQTL meta-analysis reveals differing patterns between cerebral cortical and cerebellar brain regions. *Scientific Data* 2020; **7**(1)**:** 340.
